# Mapping Hsp104 interactions using cross‐linking mass spectrometry

**DOI:** 10.1002/2211-5463.70007

**Published:** 2025-02-24

**Authors:** Kinga Westphal, Karolina Michalska, Andrzej Joachimiak, Lukasz A. Joachimiak

**Affiliations:** ^1^ Center for Alzheimer's and Neurodegenerative Diseases Peter O'Donnell Jr. Brain Institute, University of Texas Southwestern Medical Center Dallas TX USA; ^2^ Department of Medical Diagnostics, Centre for Advanced Materials and Technologies CEZAMAT Warsaw University of Technology Poland; ^3^ Center for Structural Genomics of Infectious Diseases, Consortium for Advanced Science and Engineering University of Chicago IL USA; ^4^ Structural Biology Center, X‐ray Science Division Argonne National Laboratory Lemont IL USA; ^5^ Department of Biochemistry and Molecular Biology University of Chicago IL USA

**Keywords:** Hsp104, PCSK9, protein–protein interactions, XL‐MS

## Abstract

Molecular machines from the AAA+ (ATPases Associated with diverse cellular Activity) superfamily of protein disaggregases play important roles in protein folding, disaggregation and DNA processing. Recent cryo‐EM structures of AAA+ molecular machines have uncovered nuanced changes in their conformation that underlie their specialized functions. Structural knowledge of these molecular machines in complex with substrates begins to explain their mechanism of activity. Here, we explore how cross‐linking mass spectrometry (XL‐MS) can be used to interpret changes in conformation induced by ATP in Hsp104 and how a substrate may interact with Hsp104. We applied a panel of cross‐linking reagents to produce cross‐linking maps of Hsp104 and interpret our data on previously determined X‐ray and cryo‐EM structures of Hsp104 from a thermophilic yeast, *Calcarisporiella thermophila*. We developed an analysis pipeline to differentiate between intra‐subunit and inter‐subunit contacts within the hexameric homo‐oligomer. We identify cross‐links that break the asymmetry that is present in Hsp104 in an ATP‐hydrolysis competent conformation but is absent in an ATP‐hydrolysis‐defective mutant. Finally, we identify contacts between Hsp104 and a selected protein (proprotein convertase subtilisin/kexin type 9 PCSK9) to reveal contacts on the central channel of Hsp104 across the length of this protein indicating that we might have trapped interactions consistent with its translocation. Our simple and robust XL‐MS‐based experiments and methods help interpret how these molecular machines change conformation and bind to other proteins even in the context of homo‐oligomeric assemblies enabling coupling state‐of‐the‐art modeling approaches with XL‐MS.

## Graphical abstract

This study examines how cross‐linking mass spectrometry can be utilized to analyze ATP‐induced conformational changes in Hsp104 and its interactions with substrates. We developed an analytical pipeline to distinguish between intra‐ and inter‐subunit contacts within the hexameric homo‐oligomer and discovered contacts between Hsp104 and a selected protein, PCSK9. These experiments enhance our understanding of how Hsp104 undergoes conformational changes.

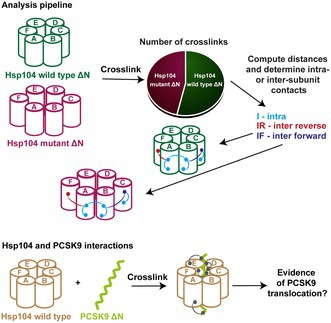

**Abbreviations**
AAA+: ATPases associated with diverse cellular activities
AB: ammonium bicarbonate
ADH: adipic acid dihydrazide
ATP: adenosine triphosphate
CD: catalytic domain
ClpB: caseinolytic peptidase B protein homolog
cryo‐EM: cryo‐electron microscopy
ct: calcorisporiella thermophila
CTD: C‐terminal domain
DLS: dynamic light scattering
DMTMM: 4‐(4,6‐dimethoxy‐1,3,5‐triazin‐2‐yl)‐4‐methylmorpholinium chloride
DSS: disuccinimidyl suberate
FDR: false discovery rate
fl: full length
FP: false positives
HPLC: high‐performance liquid chromatography
Hsp: heat shock protein
I: intra
IF: inter forward
IPTG: isopropyl β‐d‐1‐thiogalactopyranoside
IR: inter reverse
LC–MS/MS: liquid chromatography with tandem mass spectrometry
MD: middle domain
mt: mutant
NBD: nucleotide‐binding domain
NTD: N‐terminal domain
PCSK9: proprotein convertase subtilisin/kexin type 9
*R*_h_
: hydrodynamic radius
SDS/PAGE: sodium dodecyl sulfate–polyacrylamide gel electrophoresis
SEC: size exclusion chromatography
TCEP: tris (2‐carboxyethyl) phosphine
TEV: tobacco etch virus
TP: true positives
tt: thermochaetoides thermophila
wt: wild‐type
XL‐MS: cross‐linking mass spectrometry
ZL: zero length

In living organisms, molecular chaperones play important roles in folding, refolding and prevention of protein aggregation. Molecular chaperones, by interaction with their client substrates prevent their aggregation, enable them to fold properly thus retaining their functional shape [1]. Yeast heat shock protein 104 (Hsp104), along with its bacterial homolog caseinolytic peptidase B (ClpB), belongs to a AAA+ (ATPases Associated with diverse cellular Activity) superfamily of protein disaggregases that employ the energy from ATP hydrolysis to disassemble and remodel misfolded proteins [2]. Such molecular chaperones are especially important in maintaining the protein homeostasis, disturbance of which may cause several protein neurodegenerative diseases, such as Alzheimer's, Parkinson's and Huntington's [3]. In yeast, Hsp104 cooperates with Hsp70 and Hsp40 to maintain its disaggregating activity [4]. Hsp104 in yeast has been implicated in disassembling prions, including sup35 which can promote prion propagation [5]. Under normal conditions, Hsp104/ClpB concentration is rather low and significantly increases when stressful stimuli occur, which induces its ability to mediate misfolded protein disaggregation [6]. Unlike bacteria, fungi, and plants, animal cells do not encode a Hsp104 homolog which is able to promote protein disaggregation and reactivation. However, mammalian protein p97 (valosin contain protein VCP) encodes a related AAA+ molecular machine that cooperates with cofactors to recruit ubiquitinated substrates for unfolding and subsequent proteasomal degradation [7]. Recent data indicate that p97 may be implicated in disaggregation of tau and alpha‐synuclein aggregates to promote seed formation in disease [8, 9]. Thus, mechanistic knowledge on these molecular machines may yield exciting insight into control of protein disaggregation with direct implications in understanding neurodegenerative diseases.

Hsp104/ClpB is a two‐tiered ring‐shaped hexamer with an axial channel, which plays a key role in ATP‐driven translocation and renaturation of aggregates [10]. All stress‐induced molecular chaperones are divided into subfamilies based on their architecture and function (Hsp40, Hsp60, Hsp70, Hsp90, Hsp100 and small Hsp) [11]. Hsp104 is assembled into a homo‐hexameric complex that belongs to the Hsp100 family [6]. Both Hsp104 and ClpB are composed of the following domains: (a) N‐terminal domain (NTD) [12] (cooperates in substrate translocation [13]), (b) two nucleotide‐binding domains (NBD1 and NBD2), (interact with substrate and drive translocation), (c) middle domain (MD) (indispensable in substrate disaggregation and interaction with Hsp70), and (d) C‐terminal domain (CTD) (typical only for Hsp104, i.e., essential for oligomer assembly) [14, 15]. Within Hsp100, two classes of proteins can be distinguished: (a) class 1—proteins that contains two Walker‐type nucleotide‐binding domains (NBDs), like Hsp104 and ClpB and (b) class II which includes such proteins that only have one NBD domain per subunit. It is believed that two NBDs domains coordinate the binding and ATP‐drive translocation of the misfolded protein via central channel of Hsp104 hexamer and for this mode of action the presence of Hsp70 is not required [16]. These domains also contain highly conserved tyrosine residues, which form a ring that contributes to substrate binding and nucleotide‐dependent translocation [10, 17]. Such a mode of action induces conformational changes of the domains involved in translocation, which in turn causes variation in the channel diameter [12, 18]. Therefore, acquisition of high‐resolution hexameric structures of Hsp104 have been difficult, in the absence of an accurate structure it was challenging to reveal and fully understand Hsp104 mechanism of action. Advances in cryo‐EM methods have recently uncovered asymmetry in Hsp104 assemblies revealing a ‘staircase’ arrangement and a putative mechanism of substrate binding and translocation through the central pore [10, 19, 20, 21, 22]. Moreover, combined crystallographic and cryo‐EM studies on the same Hsp104 complex from *Calcarisporiella thermophila* (herein ctHsp104) revealed more nuanced conformational changes. It was determined that these two structures revealed differences in the channel width: diameter 25 to 30 Å in cryo‐EM reconstitution and 8–10 Å in the crystal filaments. Such a difference may represent two Hsp104 states: (a) the active one, with the wider channel, when the Hsp104 is ready to accept the substrate and (b) the inactive one, with a tighter channel, that prevents interaction with the substrate. Additionally, the helical rise ranges from 0 to 16 Å, which suggests that Hsp104 may adopt various stable and transient states that may be difficult to trap experimentally. It is also worth noting that Hsp104 does not require its N‐terminal and C‐terminal domain to arrange into a hexameric form [23]. However, it remains unclear how these molecular machines bind substrates that lead to subsequent unfolding and threading of the unfolded chain through the central pore.

Cross‐linking mass spectrometry yields geometric constraints between pairs of amino acid positions that provides information about the conformation and dynamics of proteins and protein–protein interactions. Combined with modeling approaches, it can provide invaluable restraints to improve *ab initio* structural models of protein complexes [24]. Despite many advantages of a XL‐MS approach, there indeed exist limitations of this approach. In dynamic systems, the cross‐linked peptides cannot unambiguously be deconvolved from the species they are derived from unless these can be biochemically isolated prior to the experiment. In the context of homo‐oligomeric assemblies, sometimes it is difficult to deconvolve intramolecular and intermolecular contacts, some of which can be resolved by analyzing interactions between heavy isotope labeled and unlabeled subunits [25]. Additionally, the identification of contacts is limited by the presence of reactive groups which have to be compatible with the cross‐linker chemistry and availability of remaining sites for subsequence proteolysis. For the study of intermolecular contacts, the data are often biased toward intramolecular contacts and often enrichment of intermolecular species is essential to improve signal of these types of interactions. Finally, post‐translational modifications, such as phosphorylation, acetylation, or ubiquitination can complicate interpretation of the mass spectrometry data as these modifications can alter proteolysis of proteins. Additionally, ionization and fragmentation of peptides may affect correct assignment of sequences to the spectra. Nonetheless, novel methods and cross‐linkers with new reactivities help circumvent these limitations allowing generation of large datasets rich with information that can be interpreted structurally using existing structural data or new modeling algorithms (i.e., alphafold).

Here, we took advantage of ctHsp104 which has been resolved using both X‐ray crystallography (PDB ID 6AZY) and cryo‐EM (PDB ID 6D00) and applied a combinatorial cross‐linking mass spectrometry approach to validate our cross‐linking data. Additionally, we use these data to interpret changes in the Hsp104 conformation due to ATP hydrolysis. Using the identified cross‐links from three different chemistries on ctHsp104 ΔN (Hsp104wt) or mutant ctHsp104ΔN2R R328M and R757M (Hsp104mt): (a) disuccinimidyl suberate (DSS), a cross‐linking chemistry that covalently bonds lysine residues (Cα‐Cα ≤ 30 Å) [26]; (b) 4‐(4,6‐dimethoxy‐1,3,5‐triazin‐2‐yl)‐4‐methylmorpholinium chloride (DMTMM)/adipic acid dihydrazide (ADH)—cross‐linking chemistry that connects carboxyl groups [27]; and (c) DMTMM and employing a novel analysis pipeline to test different spatial cross‐link geometries, we reveal how the cross‐links can be used to uncover symmetric and asymmetric interactions. Finally, we employ our XL‐MS approach to map how an associated protein (PCSK9), binds to ttHsp104 full length (Hsp104fl) and identify cross‐links that track how the substrate might traverse the pore. The data support that a cross‐linking approach and pipeline can be used to interpret conformational changes in the molecular machines and their interactions with substrates or ligands. Moreover, using this approach we can demonstrate coherence between XL‐MS data and available structures and the utilization of cross‐links from XL‐MS data may find application in reconstructing hetero‐oligomeric and homo‐oligomeric complexes. The primary goal of this study was to develop an approach that might be useful for mapping geometry of XL‐MS derived cross‐links into multi‐subunit complexes toward the goal of modeling their conformation with sparse structural information.

## Materials and methods

### Hsp104 and PCSK9 expression and purification

*Calcarisporiella thermophila* CtHsp104ΔN (153–883 aa) (Hsp104wt), ATP‐hydrolysis‐defective mutant Hsp104ΔN2R encoding R328M/R757M mutations (Hsp104mt), *Thermochaetoides thermophila* TtHsp104 full length (Hsp104fl), and PCSK9ΔN (153–692) (PCSK9) were cloned into pMCSG68 and pETM‐10 vectors. For protein sequence details, see Table S1. All details regarding protein expression and purification can be found in [23, 28]. Briefly, the gene encoding Hsp104 was amplified using PCR and cloned into pMCSG68 vector [29]. The Hsp104 double mutant (R328M/R757M) was created using modified PIPE cloning [30]. The presence of both mutations was confirmed by sequencing. The expression plasmids were transformed into *E. coli* BL21‐Gold (DE3) cells (Agilent, Santa Clara, CA, USA) and the cultures grown at 37 °C in LB‐phosphate supplemented with ampicillin. After 16 h, 30 mL of LB‐phosphate media culture of TtHsp104 full length (Hsp104fl), CtHsp104 ΔN (Hsp104wt) and Hsp104 ΔN2R encoding a R328M/R757M mutation (Hsp104mt) were added to 1 L LB‐phosphate media and grown at 37 °C, 200 r.p.m. to OD600 1.0. Next, the cell cultures were cooled to 15 °C and allowed to grow at 15 °C, 180 r.p.m. overnight. prior to induction with 0.5 mm IPTG. Cells were harvested via centrifugation (5000 ***g***, 4 °C for 20 min) and resuspended in lysis buffer (35 mL of buffer containing 20 mm HEPES‐KOH pH 7.57, 20 mm KCl (mutants) or 100 mm KCl (WT), 5% (v/v) glycerol, 20 mm imidazole pH 8.0, 2 mm MgCl_2_, 0.5 mm ATP pH 8.0, and 10 mm β‐ME). All the resuspended cells were stored at −80 °C and thawed before purification. The cells were lysed using sonication on ice (2 s bursts, followed by a 20 s pause, for 3 min) and centrifugation (20000 ***g*** for 90 min). All the samples were filtered through 0.45 μm syringe filters and purified using Ni‐NTA. Collected fractions were digested with TEV protease to remove polyhistidine tag, concentrated and purified via size exclusion chromatography (SEC) on a superdex‐200 10/300GL column. All the collected samples containing a hexamer were concentrated using 100 K concentrators and dialyzed against buffer containing 20 mm HEPES‐KOH 7.57, 20 mm KCl (mutants) or 100 mm KCl (WT), 2 mm MgCl_2_, 2 mm ATP pH 8.0 and 4 mm DTT and stored in −80 °C. DNA sequence encoding the PCSK9 was cloned from the pcDNA3.1‐F1‐WT plasmid into the pETM‐10 expression plasmid. pETM‐10‐PCKS9 and pMCSG68‐Hsp104 were transformed into *E. coli* BL21 cells and grown in LB‐phosphate supplemented with ampicillin and kanamycin. The IPTG was used to induce expression of Hsp104 and PCSK9 followed and incubated at 18 °C overnight and the complex purified as described for above for Hsp104 using Ni‐NTA, TEV cleavage and SEC.

### XL‐MS of Hsp104wt, Hsp104mt and Hsp104fl with PCSK9

Hsp104wt was diluted to 0.3 mg·mL^−1^ in 20 mm HEPES‐KOH 7.57, 100 mm KCl, 2 mm MgCl_2_, 2 mm ATP pH 8.0, and 4 mm DTT in 100 μL total volume; ctHsp104mt was diluted to 0.3 mg·mL^−1^ in 20 mm HEPES‐KOH 7.57, 20 mm KCl, 2 mm MgCl_2_, 2 mm ATP pH 8.0, and 4 mm DTT in 100 μL total volume, and Hsp104fl with PCSK9 was diluted to 0.3 mg·mL^−1^ in 20 mm HEPES‐KOH 7.57, 100 mm KCl, 2 mm MgCl_2_, 2 mm ATP pH 8.0, and 4 mm DTT in 100 μL total volume. Each sample was reacted with cross‐linkers as follows: (a) for DSS (1 mm final concentration)—1 min at 37 °C; (b) DMTMM (12 mg·mL^−1^ final concentration)—15 min at 37 °C with agitation (750 r.p.m.); (c) for ADH/DMTMM (8.3/12 mg·mL^−1^ final concentration)—15 min at 37 °C with agitation (750 r.p.m.). Reactions were quenched with 50 or 200 mm NH_4_HCO_3_ (AB) for DSS and DMTMM, ADH/DMTMM samples, respectively. Reaction was carried out at elevated temperature (37 °C for samples cross‐linked with DSS) or at room temperature (for samples cross‐linked with DMTMM, ADH/DMTMM). Even though the highest ATPase activity of ctHsp104 was determined at elevated temperature (50 °C), Hsp104 retains a significant activity at 37 °C [23].

All samples were separated electrophoretically by sodium dodecyl sulfate–polyacrylamide gel electrophoresis (SDS/PAGE) (NUPAGE™, 4–12%, Bis–Tris, 1.5 mm SDS‐Gel) and visualized by Coomassie stain to confirm cross‐link reactions proceeded to completion. After quenching, samples were lyophilized and resuspended in 8 m urea, which was followed by reduction with 2.5 mm TCEP (37 °C, 30 min) and alkylation with 5 mm iodoacetamide (RT, 30 min in darkness). Then, the samples were diluted to 1 m urea with 50 mm (AB) and trypsinized (1 : 50 m/m, Promega, Madison, WI, USA) at 37 °C (overnight, 600 r.p.m.). 2% (v/v) formic acid was added to decrease the pH of the solutions. Samples were then purified by solid‐phase extraction (Sep‐Pak tC18 cartridges, Waters®, Milford, MA, USA) and fractionated by SEC using Superdex Peptide column. The collected fractions were freeze‐dried and resuspended in water/acetonitrile/formic acid (95 : 5 : 0.1, v/v/v) to a final concentration of approximately 0.5 μg·μL^−1^. Two microliters of each sample was injected into Eksigent 1D‐NanoLC‐Ultra HPLC system coupled to a Thermo Orbitrap Fusion Tribrid system at the UTSW Proteomics core. Peptides were separated on self‐packed New Objective PicoFrit columns (11 cm × 0.075 mm I.D.) containing Magic C18 material (Michrom, 3 μm particle size, 200 Å pore size) at a flow rate of 300 nL·min^−1^ using the following gradient: 0–5 min = 5% B, 5–95 min = 5–35% B, 95–97 min = 35–95% B, and 97–107 min = 95% B, where A = (water/acetonitrile/formic acid, 97 : 3 : 0.1) and B = (acetonitrile/water/formic acid, 97 : 3 : 0.1). The mass spectrometer was operated in data‐dependent mode by selecting the five most abundant precursor ions (m/z 350–1600, charge state 3+ and above) from a preview scan and subjecting them to collision‐induced dissociation (normalized collision energy = 35%, 30 ms activation). Fragment ions were detected at low resolution in the linear ion trap. Dynamic exclusion was enabled (repeat count 1, exclusion duration 30 s).

The analysis of the mass spectrum data was done by xQuest [31]. For each cross‐link dataset Hsp104wt (DSS, ADH_DMTMM and DMTMM), Hsp104mt (DSS, ADH_DMTMM and DMTMM), we employed the manually modified *Calisporella thermophila* Hsp104 sequence (uniprot A0A452CSQ7) encoding ΔNHsp104wt or ΔNHsp104mt as well as *Thermochaetoides thermophila* encoding full length Hsp104 (uniport A0A2Z6G185) and human PCSK9 (uniprot Q8NBP7) missing the first 152 residues—(see Table S1) to identify true positives (TPs). To identify false positives (FPs), the reverse sequence was searched in parallel.

Each raw data were first converted to open.mzXML format using mscovert (proteowizard.sourceforge.net). Search parameters were set differently based on the cross‐link reagent. For DSS cross‐link search, maximum number of missed cleavages (excluding the cross‐linking site) was 2, peptide length was 5–50 aa, fixed modifications were carbamidomethyl‐Cys (mass shift = 57.021460 Da), mass shift of the light cross‐linker was 138.068080 Da, mass shift of monolinks was 156.078644 and 155.096428 Da, MS1 tolerance was 10 ppm, and MS2 tolerance was 0.2 Da for common ions and 0.3 Da for cross‐link ions, search in ion‐tag mode. For DMTMM zero‐length cross‐link search, maximum number of missed cleavages was 2, peptide was 5–50 residues, fixed modifications were carbamidomethyl‐Cys (mass shift = 57.02146 Da), mass shift of cross‐linker was − 18.010595 Da, no monolink mass specified, MS1 tolerance was 15 ppm, and MS2 tolerance was 0.2 Da for common ions and 0.3 Da for cross‐link ions, search in enumeration mode. For ADH, maximum number of missed cleavages (excluding the cross‐linking site) was 2, peptide length was 5–50 residues, fixed modifications were carbamidomethyl‐Cys (mass shift = 57.021460 Da), mass shift of the light cross‐linker was 138.09055 Da, mass shift of monolinks was 156.10111 Da, MS1 tolerance was 15 ppm, and MS2 tolerance was 0.2 Da for common ions and 0.3 Da for cross‐link ions, search in ion‐tag mode. False discovery rates (FDRs) were estimated using xprophet [32] to be 0.0–0.46 and were calculated as TP/(FP + TP) at a given Id‐score cutoff. For Hsp104wt and Hsp104mt, the data were trimmed between Id‐score 16–22. For Hsp104fl:PCSK9, ADH_DMTMM and DMTMM were trimmed at Id‐score 15. For Hsp104fl:PCSK9 dataset, we excluded DSS cross‐linking results due to the fact that all data had an unsatisfactory Id‐scores.

### Measurement of hydrodynamic properties

To measure the value of hydrodynamic radius, we employed dynamic light scattering (DLS), which measures fluctuations in the intensity of scattered light from particles in solution [33]. For this purpose, we prepared Hsp104wt and Hsp104mt samples (25 μL; 1 mg·mL^−1^). Samples were filtered through a 0.22 μm PES sterile filter and loaded onto a 384 well clear flat‐bottom plate. Measurements were performed using Wyatt DynaPro Plate Reader III. For the data analysis, we employed wyatt dynamics software version 7.8.2.18. For comparison, we also calculated the radius of gyration of Hsp104 using hydropro [34].

### Interpretation of cross‐links on experimental structures of Hsp104

To validate the identified cross‐links, we employed strict score‐ID cutoffs (≥ 16) enabling identification of a larger number of cross‐links that using standard metrics yields higher FDRs but the TPs can be ultimately validated on an experimental ground truth structure. Our analysis highlights that classical FDR‐based cutoffs overestimate errors but are required in experimental setups with many unknowns. By contrast, in simplified systems with available structural information, TP hits can be validated ultimately leading to richer datasets that can capture more dynamic and transient interactions. To map cross‐links on structures, Cα‐Cα Euclidean distances between cross‐linked positions were calculated using xwalk [35] using subunit and subunit‐subunit geometries derived from ctHsp104 structures determined using X‐ray and cryo‐EM (PDB IDs 6D00 and 6AZY, respectively). For DSS, ADH/DMTMM, and DMTMM XL‐MS datasets, we used established Euclidean Cα‐Cα distance cutoffs for DSS (30 Å), ADH/DMTMM (21 Å) and DMTMM (15 Å) as described previously [36, 37]. The distances between cross‐linked positions were evaluated as intramolecular (within subunits A, B, C, D, E, and F) and two forms of intermolecular that are dependent on the order of the positions: (a) inter‐subunit forward (AB, BC, CD, DE, and EF) and (b) inter‐subunit reverse (BA, CB, DC, ED, and FE). Since each cross‐link can be formed within one subunit or between different configurations of neighboring subunits, we employed heat maps to order the distances and determine which cross‐links (intramolecular, inter‐subunit forward, or inter‐subunit reverse—for detail, see Fig. 2) were acceptable given an established geometric distance cutoff consistent with the cross‐linker chemistry and side chain flexibility. For Hsp104fl:PCSK9 complex, only residues that formed intermolecular cross‐links (i.e., between Hsp104 and PCSK9) were mapped onto Hsp104fl structure to determine which protein region predominantly interacts with the substrate. All the cross‐link mappings were visualized using pymol, Schrodinger, New York, NY USA, while the figures were prepared using custom Gnuplot scripts, graphpad prism 9.4.1, Dotmatics, Bishop's Stortford, UK, and Adobe Illustrator.

## Results

### Strategy to interpret XL‐MS data on experimentally determined structures of Hsp104

**Fig. 1:**
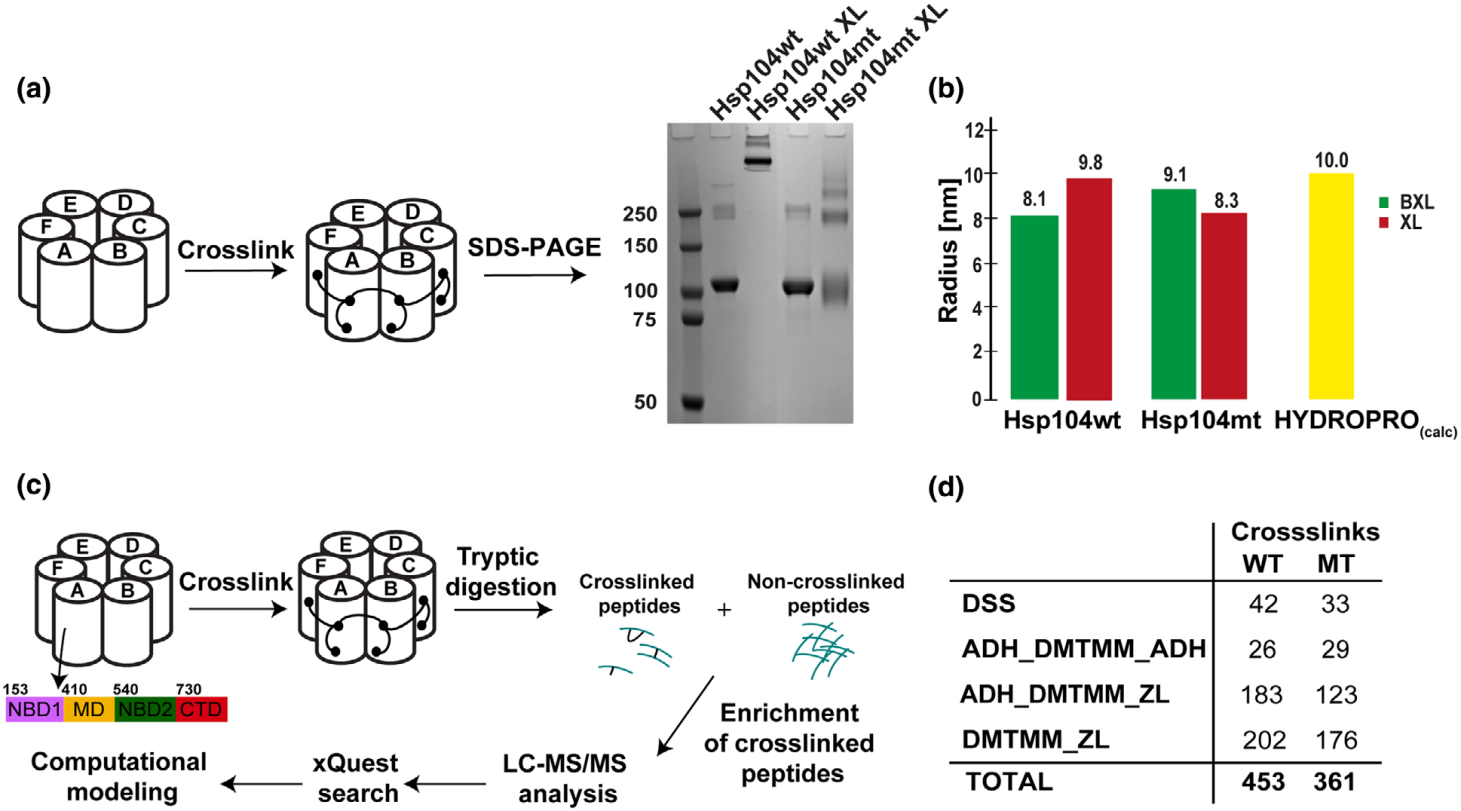
Mass spectrometry analysis of cross‐linked Hsp104. (a) Cross‐linking of Hsp104wt (wild‐type) and Hsp104mt (mutant) leads to the formation of higher molecular species that approaches the hexameric assembly. (b) Hsp104wt/mt assemblies exhibit defined hydrodynamic radii as measured by dynamic light scattering (DLS) in the absence [green—BXL (before cross‐linking)] and presence of cross‐linker [red—XL (after cross‐linking)] and are consistent with calculated radii derived from experimentally determined structures of Hsp104wt (PDB ID 6D00). (c) Both Hsp104wt and Hsp104mt were reacted using the following cross‐linkers: (i) DSS (disuccinimidyl suberate), (ii) ADH (adipic acid dihydrazide) and DMTMM (4‐(4,6‐dimethoxy‐1,3,5triazin‐2‐yl)‐4‐methylmorpholinium chloride); (iii) DMTMM. For that purpose, Hsp104wt/Hsp104mt was treated with the particular cross‐linker and enzymatically digested to yield a pool of cross‐linked and noncross‐linked peptides. All cross‐linked peptides were chromatographically enriched and analyzed using liquid chromatography with tandem mass spectrometry (LC–MS/MS). The identification of the peptides and linked (affected) chains was performed with the use of xQuest (see Materials and methods). (d) The number of all cross‐links formed in Hsp104wt/Hsp104mt after treatment with particular cross‐linker. Experiments were performed as one (*n* = 1) biologically independent replicate.

**Fig. 2:**
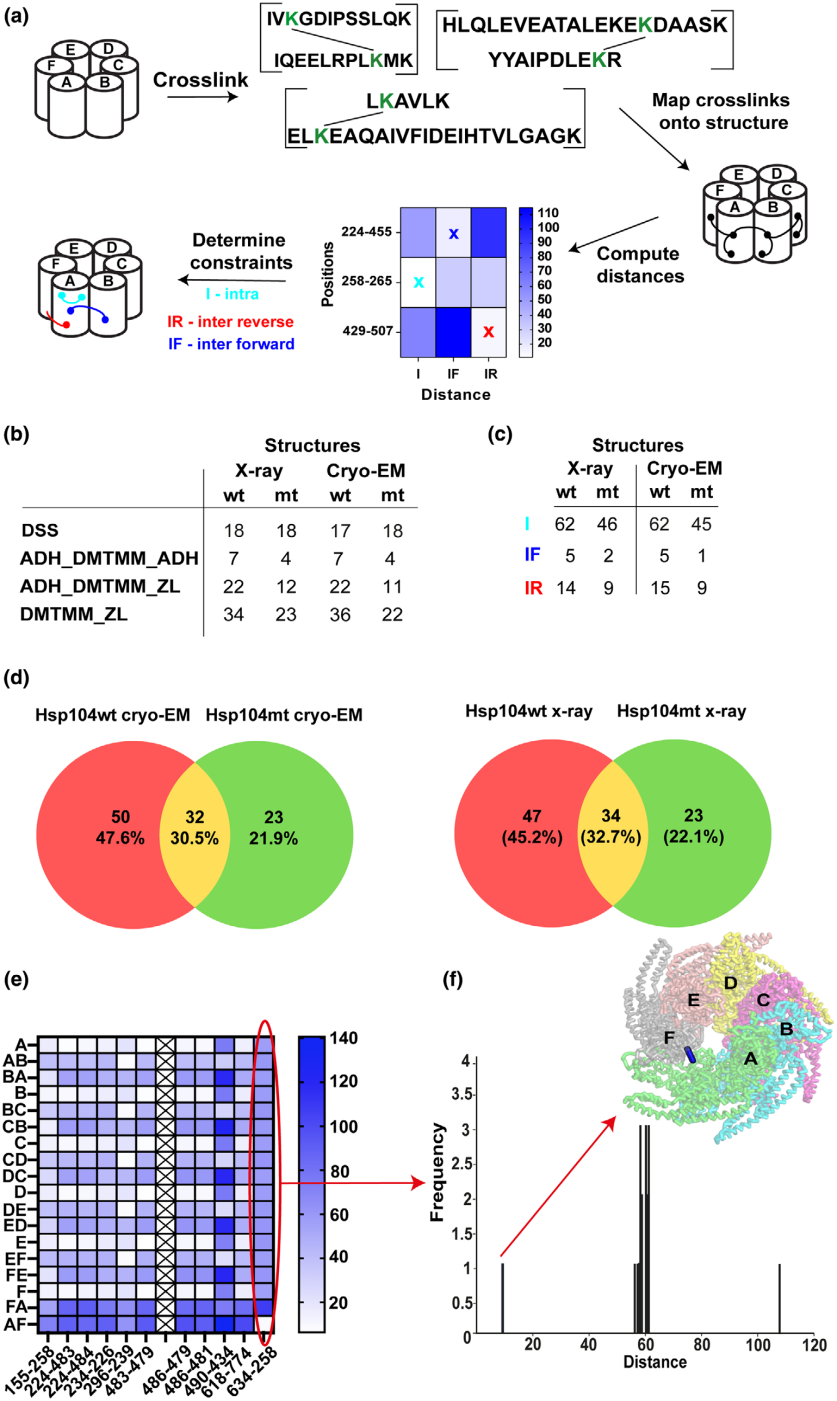
Analysis of Hsp104wt/mt (wild‐type/mutant) mass spectrometry derived cross‐links on experimental structures of Hsp104. (a) Using different cross‐linkers, we employed our pipeline to identify cross‐links between peptides (e.g., formed between lysine‐lysine, acidic–acidic, or lysine‐acidic residues). Cross‐links were mapped onto Hsp104 X‐ray and cryo‐EM (cryo‐electron microscopy) (cryostructures, PDB IDs 6D00 and 6AZY, respectively) and the C‐C distances (in Å) between cross‐linked positions were computed using Xwalk (see Materials and methods). We assessed the compatibility of the identified cross‐links in different subunit and subunit‐subunit geometries discriminating between intra‐subunit cross‐link (I) (formed within one subunit) or inter‐subunit cross‐link (formed between two neighboring subunits) that fall into two subtypes (dependent on the order of the positions), whether it was formed clockwise [inter‐subunit forward (IF)] or counter‐clockwise [inter‐subunit reverse (IR)] (see Materials and methods for full description). All distance values (in Å) were grouped using heat maps—for each restraint three distance values corresponding to I, IF, and IR cross‐links were included. The brighter the color, the shorter the distance between two linked residues. In each case, the shortest distance value determined the type of cross‐link, which might be I, IF, or IR, respectively. The other cross‐links, which distance values exceeded the admissible value were excluded from cross‐linking data. (b) Summary of cross‐links formed in Hsp104wt/Hsp104mt after treatment with particular cross‐linker, which distance values are acceptable. The results revealed the similarities between X‐ray and cryo‐EM structures. As it can be noticed, the number of cross‐links formed onto Hsp104wt cryo‐EM and X‐ray structure as well as Hsp104mt cryo‐EM and X‐ray structure is comparable. (c) Summary of cross‐link types formed in Hsp104wt/Hsp104mt after treatment with a particular cross‐linker. These data also confirm that cryo‐EM and X‐ray structures are similar. The number of subtypes of cross‐links formed onto Hsp104wt cryo‐EM and X‐ray structure as well as Hsp104mt cryo‐EM and X‐ray is almost the same. (d) Overlap of all the cross‐links identified in each of the two (Hsp104wt and Hsp104mt) datasets. (e) With the use of heat maps, it was possible to find distinctive cross‐links based on calculated distances (in Å). In this case, the cross‐links formed between 634/637 and 258 residues of A and F units of Hsp104 fits perfectly the structure (the 634–258 is presented as a representative). (f) The representative 634 (A)‐258 (F) contact that fits onto Hsp104 subunits (distance < 20 Å). All other geometries within subunits or between subunits lead to distances larger than 55 Å. Each of the protein subunits has its own color to facilitate their identification. Experiments were performed as one (*n* = 1) biologically independent replicate.

The molecular chaperone Hsp104 is comprised of six identical subunits arranged in a ring. To gain molecular insight into the structure and asymmetry of the assembly, we compared wild‐type CtHsp104ΔN (153‐883 aa) (herein Hsp104wt) and mutant CtHsp104ΔN2R encoding the R328M/R757M mutations (herein Hsp104mt) unable to hydrolyze ATP (Table S1) [38]. The purified Hsp104wt and Hsp104mt complexes were confirmed to be hexameric using SEC (Table S2). We next employed single‐residue resolution cross‐linking mass spectrometry approach (Fig. 1c) leveraging several different chemistries, including DSS, ADH/DMTMM, and DMTMM alone on Hsp104wt and Hsp104mt. These cross‐linkers enable trapping specific contacts between lysine residues (DSS), acidic residues (DMTMM/ADH) and between acidic and lysine residues (DMTMM), respectively [36, 37]. Cross‐linked reactions of Hsp104wt and Hsp104mt were resolved by SDS/PAGE to confirm that addition of a cross‐linker can trap covalent interactions between subunits within the hexameric assembly (Fig. 1a). Interestingly, cross‐linking reactions with Hsp104wt lead to defined species consistent with a uniform hexamer while for the Hsp104mt we observe a ‘laddering’ effect suggesting that we can trap larger assemblies but also intermediates, consistent with this mutant adopting a more dynamic hexamer conformation. We also used DLS to measure the hydrodynamic radius (*R*_h_) of Hsp104wt and Hsp104mt samples before and after cross‐linking to confirm the physiological conformation of the samples. For both Hsp104wt and Hsp104mt before and after cross‐linking, the measured *R*_h_'s are estimated to be around 10 nm, which is consistent with estimation of *R*_h_ from experimentally determined structures of Hsp104wt [23] using hydropro [34] (Fig. 1b). After applying standard score‐ID cutoffs for cross‐linking reactions using DSS, ADH/DMTMM and DMTMM, we cumulatively identified 453 cross‐links for Hsp104wt datasets while and 361 for the Hsp104mt dataset (Fig. 1d, see Materials and methods for details), which could be explained by differences in the dynamics between Hsp104wt and Hsp104mt. For the Hsp104 from this species, there are two structures available that were determined by X‐ray crystallography and cryo‐EM and revealed a left‐handed helical assembly with unprecedented atomic detail [23]. We next decided to interpret our large cross‐linking dataset using these two experimentally determined structural models. While these Hsp104 assemblies are homo‐oligomeric, there are three possible cross‐link geometries between a pair of residues: intra‐subunit (intramolecular; I), inter‐subunit forward (IF), and inter‐subunit reverse (IR) (Fig. 2a) when looking at three contiguous subunits (Fig. 2a—subunits F, A, and B). Furthermore, if we assume any asymmetry between the subunits, we must consider six possible intra, inter‐forward and inter‐reverse geometries.

### Mapping identified cross‐links onto discrete subunit geometries

We set out to interpret our comprehensive XL‐MS dataset derived from three different cross‐linking chemistries on the available X‐ray crystallography and cryo‐EM structures of ctHsp104 (Fig. 1c) [23]. At first, for both Hsp104wt and Hsp104mt, the data were trimmed at score IDs ≥ 16–21 to maximize the number of TPs (see Fig. S1 and Materials and methods) reducing the number of cross‐links to 146 for Hsp104wt and 86 for Hsp104mt for subsequent validation on the experimental structures. These cross‐links were mapped onto all possible combinations of geometries for a single subunit (I, i.e., A, B, C, and D) and two geometries between subunits, inter‐subunit forward (IF, i.e., AB, BC, and CD) and inter‐subunit reverse (IR, i.e., BA, CB, and DC). This calculation was performed for all single subunits and all local subunit pairs (A, AB, BA, B, BC and CB, etc…) using the Xwalk software computing Euclidean Cα‐Cα distances [35]. We first ascertained how well our identified cross‐linked pairs fit on each structure by calculating the Cα‐Cα distance for all possible subunit geometries (e.g., I, IF, and IR) initially focusing on the X‐ray structure. Since each residue can form a cross‐link with a second residue within a subunit (I) or neighboring subunit (IR or IF), it was essential to first classify all cross‐links and identify the most probable geometry based on distance (for details, see Fig. 2a). Using this strategy, we were able to map 81 cross‐links onto Hsp104wt and 57 cross‐links onto Hsp104mt X‐ray structures (see Fig. 2b). To simplify interpretation of this data, we used heat maps to order all the distance values (see Figs S2–S5), thus enabling precise assignment of contacts based on subunit and cross‐linker geometry to those shorter than 30 Å (for DSS), 21 Å (for ADH_DMTMM_ADH), and 15 Å (for ADH_DMTMM_ZL and DMTMM_ZL). All identified cross‐links were mapped onto both X‐ray and cryo‐EM structures. This enabled the assignment of probable subunit or subunit‐subunit geometries for all the cross‐links that mapped onto the X‐ray structure of ctHsp104 for the Hsp104wt (75 cross‐links) and Hsp104mt (49 cross‐links) XL‐MS datasets (see Fig. S8). The numbers of cross‐links were comparable when mapped onto the cryo‐EM structure (Fig. 2b, Table S3 and Fig. S9). Of the cross‐links that were interpretable on the structures, 73% to 100% of all cross‐links were consistent with the geometric cutoffs for each chemistry (for details, see Table S3). These values are consistent with our calculated FDRs (see Fig. S1). For the zero‐length cross‐link dataset, we included cross‐links with lower Id‐scores which inflated the FDR values, but enabled inclusion of a larger number of cross‐links that we could validate on the experimental structures. Despite higher FDRs, the majority of the cross‐links can be validated using the experimental Hsp104 structure. Moreover, we gained more cross‐links that we can validate on the experimental structure for the Hsp104wt dataset compared with Hsp104mt (see Fig. S7a,c). We illustrate this on the ADH_DMTMM_ZL Hsp104wt cross‐linking dataset. At an FDR of 34%, we obtain 30 cross‐links, of which 22 can be validated on the structure, leading to 73% of the cross‐links consistent with the structure and is higher than the estimated FDR. Reducing the FDR to 11%, all five cross‐links are validated but 13 of the 17 gained are not included leading to a loss of information—this suggests that the error models do not correctly estimate FDRs. Similarly, in the Hsp104wt DMTMM_ZL dataset, when applying an FDR threshold of 41%, 36 of the 50 total identified cross‐links can be validated on the structure. Reducing the FDR to 9% decreases the number of validated cross‐links to 28 out of 37 contacts. In both of these cases, over 70% of cross‐links can be validated using the structure outperforming the FDR estimates. Reduction in the FDR does indeed enable all identified cross‐links to be validated, but it reduces the datasets dramatically loosing valuable information. This highlights the importance of available experimental structures (or reliable models) to validate the detected contacts and furthermore helps identify contacts that may be rare, thus harder to detect, but yet physiologically important for the protein complex. We illustrate this by comparing the changes in the number of cross‐links as a function of FDR (Fig. S7a). Interestingly, using the structure to validate the cross‐links at two different FDR values does not dramatically reduce the percentage of cross‐links that can be validated on the experimental structure, highlighting the power of having an accurate structural model for the molecular system being tested in the experiments. Importantly, using experimental structures to validate high ‘FDR’ cross‐links enables gains in all the cross‐links that might be relevant, regardless of the nature of the interaction (transient or stable) or their abundance. Finally, the X‐ray and cryo‐EM structure used for mapping is derived from the wild‐type Hsp104, which might explain why the number of cross‐links that we can validate Hsp104wt is much higher than for the Hsp104mt dataset and again highlights how knowledge of conformational changes in response to mutations may not be easy to predict in the absence of direct structural information.

**Fig. 3:**
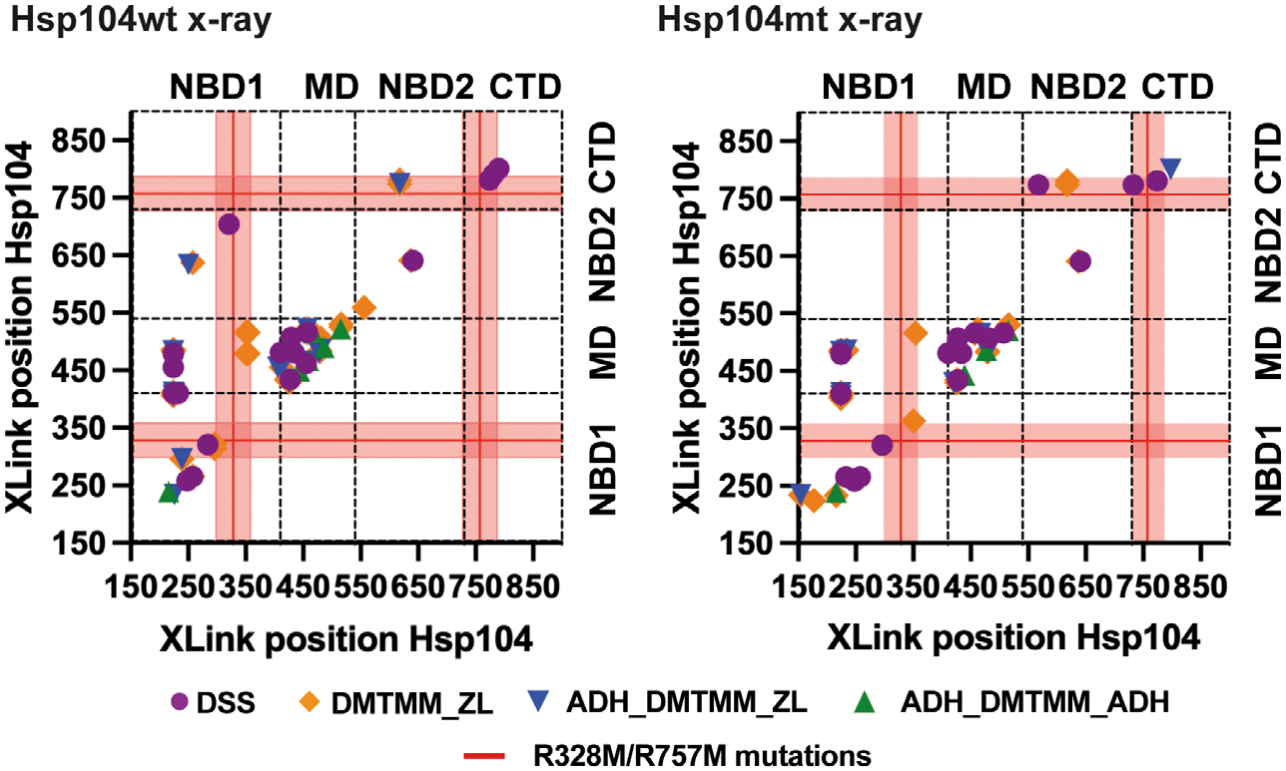
Hsp104 domain distribution of Hsp104wt (wild‐type) and Hsp104mt (mutant) cross‐links across chemistries that are consistent with the Hsp104 X‐ray structure. Cross‐linked pairs plotted to show connectivity of contacts within and across domains from the Hsp104wt/mt XL‐MS (cross‐linking mass spectrometry) datasets showing only contacts that were consistent with the experimental X‐ray Hsp104 structure. The largest number of cross‐links was formed within NBD1 (nucleotide‐binding domain) and MD (middle domain) domains. Only minor differences can be detected between Hsp104wt and Hsp104mt (Hsp104mt forms fewer cross‐links). In regions proximal to the mutated residues in Hsp104mt, less cross‐links were detected in Hsp104mt (7 cross‐links) compared with Hsp104wt (10 cross‐links) as defined as 328 ± 30 and 757 ± 30 (red lines indicate sites of mutation and colored area includes region considered as hits). Cross‐links are colored by chemistry and are shown in purple, orange, blue, and green for DSS, DMTMM_ZL, ADH_DMTMM_ZL, and ADH_DMTMM_ADH, respectively. Domains are separated by dashed lines. For cryo‐EM (cryo‐electron microscopy) variants see Fig. S6.

Of all the cross‐linking chemistries tested, the highest number of cross‐links were observed in the DMTMM reactions, which traps zero‐length cross‐links between lysine residues to carboxylic groups of glutamic or aspartic acid for which we identified 34 and 23 cross‐links that are interpretable on the X‐ray structure (see Fig. 2b—the data include all mapped cross‐links, without distance cutoff). From our calculations, we also partitioned the contacts into I, IR, and IF to determine which contacts are the most frequent in our dataset. The data revealed that intramolecular cross‐links within a subunit constitute the most common type of linker, in both cases intra cross‐links represent around 80% of all cross‐links formed in Hsp104wt and Hsp104mt. The rest of the cross‐links were formed between two neighboring units (see Fig. 2c). We also find that the majority of intramolecular cross‐links are localized to the MD and NBD1 domains (see Fig. 3 and Fig. S6). It is perhaps not surprising that intramolecular contacts are the most abundant contacts as these should be the least dynamic. Partitioning of the IR and IF inter‐subunit contacts is less interesting because it is purely derived from the ordering of the pairs from the rather arbitrary assignment of the two peptides. The assignment of the first (A) and second (B) peptides in the software where peptide A–peptide B contacts can be attributed to the IR inter‐subunit but are equivalent to peptide B–peptide A in the IF inter‐subunit arrangement. It also has to be emphasized that we compared regions containing mutated residues (R328M and R757M). We determined that within Hsp104wt R328M (±30 residues) region eight cross‐links are formed (284–321; 296–322; 296–314; 354–479; 351–479, 516–351; 516–354, and 321–704), while in the same region of Hsp104mt sequence, only three cross‐links were detected (296–321; 354–516 and 363–350) (Fig. 3). This may suggest that the Hsp104wt active site may be more ordered because it is bound to ATP and in turn, may provide further evidence that the Hsp104mt does not bind ATP and as a result it reduces the efficiency of cross‐linking reaction. Additionally, we determined that within Hsp104wt R757M (±30 residues) region four cross‐links are formed (781–774; 790–781, 618–774, and 618–781) as well as in the same region of Hsp104mt sequence we also detected four cross‐links (733–774; 781–774; 618–774 and 618–781). While there is a 19% decrease in overall number of cross‐links for Hsp104mt compared with Hsp104wt, the domain distribution of cross‐links pairs is similar between the two datasets. Furthermore, comparing the cross‐links that can be explained on the cryo‐EM and X‐ray structures show high similarity (Fig. 3 and Fig. S6). Together, our data and analysis suggest that we can evaluate our cross‐linking data in the context of experimental structures even when applied to homo‐oligomeric assemblies enabling assignment of specific subunit geometries. All the other cross‐links that fit onto Hsp104 structures are presented in Fig. S11. We would like to emphasize that the inclusion of high FDR cross‐links must be interpreted with caution, in particular, if structural information is unavailable.

### Identification of XL‐MS contacts consistent with asymmetric subunit geometry

Comparing the data obtained for the Hsp104wt and Hsp104mt, we found that the wild‐type complex yielded more cross‐links and the percentage of cross‐links consistent with the Hsp104 structures (both X‐ray and cryo‐EM). It is also possible that the Hsp104mt conformation is more dynamic, which can decrease the likelihood of the formation of cross‐links within the permissible distance range less probable compared with Hsp104wt observed in the experimental structures. Additionally, Hsp104wt is able to hydrolyze ATP and thus could represent a lower energy posthydrolysis state. Indeed, changes in dynamics in response to ATP hydrolysis have been previously noted in other molecular machines, such as the eukaryotic chaperonin TRiC/CCT [37]. To discover other differences between Hsp104wt and Hsp104mt based on XL‐MS patterns, we compared the overlap of all the cross‐links identified in each of the two datasets and find that 34 cross‐links are the same across the two datasets with only 47 and 23 different for Hsp104wt X‐ray and Hsp104mt X‐ray, respectively (Fig. 2d). Similar values were obtained for Hsp104wt/Hsp104mt cryo‐EM—32 cross‐links are the same, while 50 and 23 are different, respectively. Employing a higher FDR but cross‐validating the cross‐links on an experimental structure, we were also able to identify two unique cross‐links that are present only in the Hsp104wt XL‐MS data. These two cross‐links (634–258 and 637–258) may indicate asymmetry within the Hsp104 hexamer, as they exclusively match the asymmetric subunit pairing between F and A. These contacts are incompatible with the other subunit geometries (i.e., AB, BC, CD, DE, and EF) where the distances are larger but because the helical twist begins at subunit A and ends on subunit F (i.e., defined by the ‘staircase’ arrangement of the hexamer) the spacing between these two subunits is smaller (see as an example Fig. 2e,f), where the Cα‐Cα distance between cross‐linked residues, 634 and 258, equals 6.5 Å, while for other subunit pairs, the Cα‐Cα distances are large ranging between 60 and 100 Å. Importantly, these cross‐links in both cases (ADH_DMTMM_ZL and DMTMM_ZL) arise from the connection between residue 258 and either residue 637 or 634 (see Fig. S10). It may suggest that this region is unique, especially in the context of the spatial arrangement of subunits A and F, as in all other cases, the distance between these residues significantly exceeds the permissible maximum distance for amino acid residue contacts using zero‐length cross‐linkers. It may also explain their low Id‐scores, as due to their rare abundance they may be more transient. For the Hsp104mt dataset, we also did not observe the formation of similar cross‐links between subunits A and F. Furthermore, the cross‐link distances were consistent with the cross‐linker geometry in both the cryo‐EM and X‐ray structures (Figs S8 and S9), which confirms that these two structures, although determined by different techniques, are similar to the conformation in solution used in the XL‐MS analysis. Because these cross‐links were not identified in the Hsp104mt cross‐link dataset, it could indicate that the ATP‐hydrolysis‐defective mutant may not be able to sample these FA contacts as readily. Thus, our data suggest that we have been able to identify a unique interaction that is sampled more frequently (i.e., stably) in the Hsp104wt complex but less frequent in the Hsp104mt. We suspect that this is largely driven by ATP‐hydrolysis activity which may rigidify the assembly while the ATP‐hydrolysis‐defective mutant is more dynamic.

**Fig. 4:**
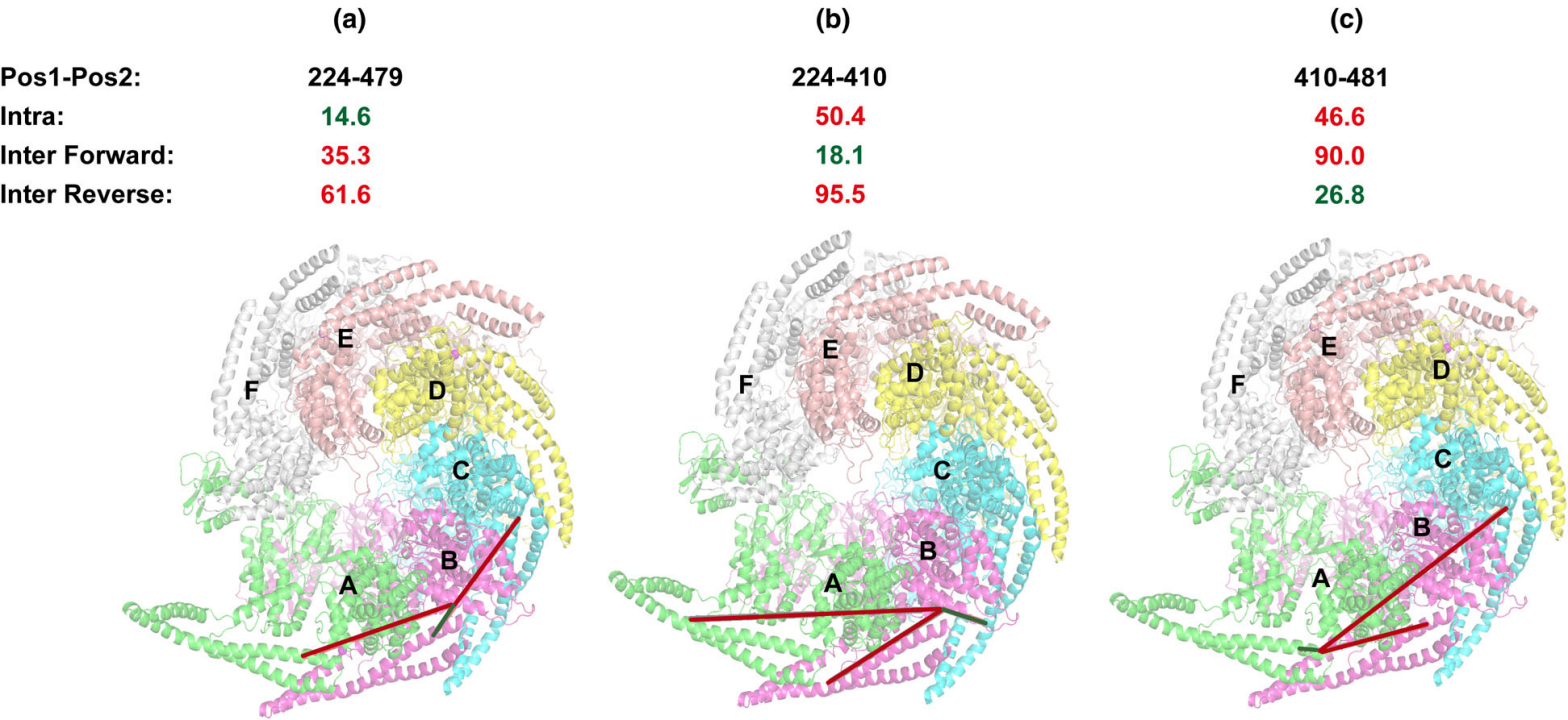
Geometry‐based strategy to discriminate geometrically compatible cross‐link. The selected cross‐links were mapped onto Hsp104 structure as a representative of each type of cross‐link. The cross‐links were mapped onto the structure in each configuration: (a) within B unit, which corresponds to intra (I) cross‐link, (b) between B and C units, which represents the intra forward (IF) linkage, and (c) between B and A units, which depicts the inter‐reverse (IR) cross‐link. On the left side, the presented cross‐link formed acceptable linkage only within B unit (green lines), while BC and BA distances were too long and unacceptable (red lines). In the exact same way, the middle structure and the right structure present the formation of inter‐forward (IF) and inter‐reverse (IR) cross‐link, respectively.

To highlight how a contact fits one specific subunit geometry over others, we illustrate this visually by mapping the distances on a subunit pair (i.e., AB) (Fig. 4). The cross‐links for which distances are longer than the permissible distance (based on geometry of the cross‐linker) were presented as red lines while the cross‐links below the acceptable distance threshold were presented as a green line. In the case of the 224–479 cross‐link, the computed distances for intra (within B), inter‐forward (BC), and inter‐reverse (CB) geometries confirmed that the cross‐link distance value is only acceptable for interaction within B unit—distances of BC and CB cross‐links exceeded the assumed limit value. For the 224–410 cross‐link, only BC linkage was acceptable (Fig. 4b), while the cross‐link 410–481 represents the formation of inter‐reverse cross‐link (CB) (Fig. 4c).

### Probing Hsp104‐associated protein interactions by XL‐MS

**Fig. 5:**
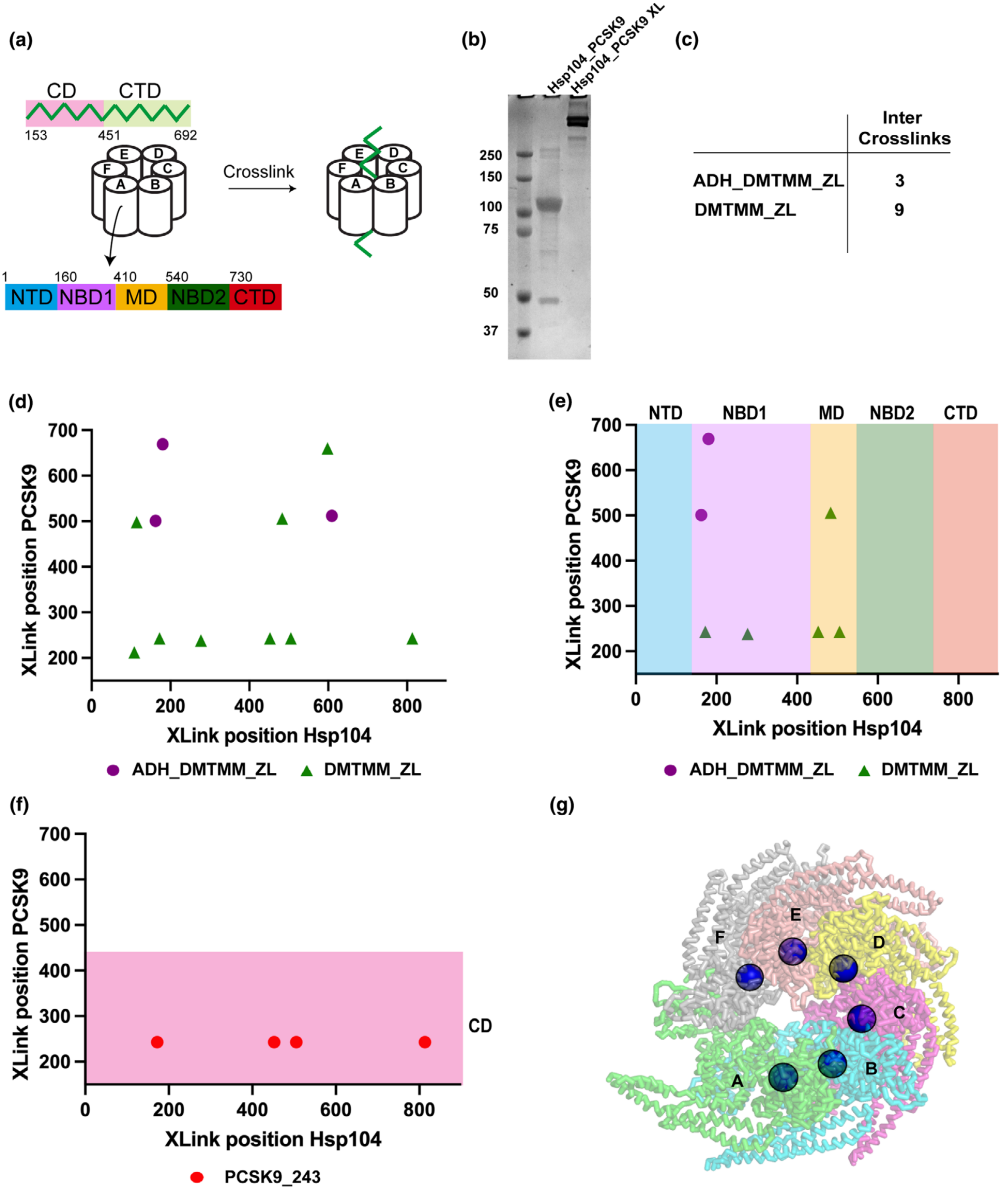
Hsp104 full length (Hsp104fl) interacts with PCSK9 (proprotein convertase subtilisin/kexin type 9). (a) Hsp104fl was cross‐linked with PCSK9 using commercially available cross‐linking agents to determine its protein regions that interact with a substrate since Hsp104 is an ATP‐dependent molecular machine that facilitates the disaggregates refolding. To reveal which region predominately binds the substrate, a schematic arrangement of the Hsp104fl and PCSK9 domains are shown. PCSK9 domains; catalytic domain (CD), C‐terminal domain (CTD). Hsp104fl domains: N‐terminal domain (NTD), middle domain (MD), NBD1 and NBD2—nucleotide‐binding domains 1 and 2, and C‐terminal domain (CTD). It is believed that Hsp104 hexamer is active against aggregates which bind in the axial channel of the chaperone, hence it is essential to determine residues that cross‐link to misfolded protein. (b) Cross‐linking reaction of Hsp104fl:PCSK9 leads to the formation of higher molecular species, which was confirmed by SDS/PAGE analysis. The disappearance of bands corresponding both to Hsp104fl and PCSK9 also provides an indirect confirmation of the interaction between Hsp104fl and PCSK9. (c) Hsp104fl and PCSK9 were cross‐linked with ADH (adipic acid dihydrazide) and DMTMM (4‐(4,6‐dimethoxy1,3,5‐triazin‐2‐yl)‐4‐methylmorpholinium chloride). As a result, three inter cross‐links were generated using the mixture of ADH and DMTMM, and nine inter cross‐links were detected after cross‐linking with DMTMM. (d) All inter cross‐links formed between Hsp104fl and PCSK9 are presented in the chart. In this case, positions of amino acids that form a cross‐link are plotted against each other to reveal Hsp104fl and PCSK9 regions that are involved in cross‐linking formation. The data plotted vertically represent residues of Hsp104fl that form the particular cross‐link, while those plotted horizontally correspond to PCSK9 amino acids. Each cross‐linking chemistry was marked with its symbol: (i) ADH_DMTMM_ZL (zero length) is a purple circle and (ii) DMTMM_ZL is a green triangle. (e) In Hsp104fl protein, the largest possible number of cross‐links was formed within its NBD1 and MD domains. (f) In PCSK9, the largest number of cross‐links was formed within catalytic domain; however, each of the domains exhibited ability to interact with Hsp104fl. Especially, 243 residue is an active chain that forms four cross‐links with Hsp104fl residues. This may suggest that PCSK9 is translocated through various Hsp104fl residues, included those localized in centrally placed channel of the Hsp104fl structure. (g) Residue that confirms the binding of PCSK9 in axial channel of Hsp104fl. Experiments were performed as one (*n* = 1) biologically independent replicate.

The recent cryo‐EM revolution has yielded an enormous number of structures of molecular machines [39, 40, 41, 42, 43, 44, 45, 46]. For many years, these machines, including Hsp104, were modeled using symmetric averaging but new developments in instrumentation and methods have allowed reconstruction of asymmetric structures in different conformations [23, 47]. Additionally, these structures have begun to uncover how substrates may bind to Hsp104 and thread through the pore [22]. PCSK9 is a human protease that is involved in processing low‐density lipoprotein receptors to regulate low‐density lipoprotein cholesterol [48]. PCSK9 is normally insoluble when expressed recombinantly in bacteria [23]; however, when PCSK9 is recombinantly co‐expressed with Hsp104fl, PCSK9 can be solubilized and a complex between Hsp104 and PCSK9 can be isolated. SEC analysis of this complex uncovers molecular weights consistent with an Hsp104fl hexamer bound to a single PCSK9 (Table S2). Here, we attempted to leverage high‐resolution XL‐MS to trap intermediates of an associated protein, PCSK9 ΔN (PCSK9), bound to full length *Thermochaetoides thermophila* Hsp104 (Hsp104fl). The complex was reacted with DMTMM/ADH and DMTMM and as previously we employed SDS/PAGE (Fig. 5b) to confirm the formation of higher molecular species, which was followed by samples processing according to our XL‐MS pipeline (Fig. 5a). We identified 12 intermolecular cross‐links formed between Hsp104fl and PCSK9 (Fig. 5c and Table S4). The majority of the cross‐links identified were derived from the DMTMM reactions (Fig. 5d, green triangle) and moreover almost each region (i.e., domain) of Hsp104fl formed cross‐links with PCSK9. We also determined that Hsp104 NBD1 (four cross‐links) and MD (three cross‐links) are mainly involved in the interaction with the model substrate—residues within these two regions bind to different amino acids of PCSK9 (Fig. 5e). Additionally, we find that the CD (six cross‐links) of PCSK9 is more frequently cross‐linked to Hsp104fl. We determined that residues from CD of PCSK9 are cross‐linked with each domain of Hsp104fl. It may confirm that initial recruitment of substrate to surfaces begins on the outside of Hsp104fl as this domain has been implicated in binding to substrates using biochemical approaches and structural approaches [21, 49]. Similarly, in the PCSK9 sequence, we determined that residue 243 interacts with several Hsp104fl positions (Fig. 5f and Fig. S13). The 243 position was cross‐linked to four different amino acids of Hsp104fl, which suggests that entire polypeptide has an ability to translocate through various chaperone surfaces, including these residues located in axial channel that provides its disaggregating activity (Fig. 5g). This also suggests that the initial encounter complex is mediated by N terminus not the C terminus. It has to be emphasized that in this experiment, we also included cross‐links with lower Id‐score (see Fig. S12). It might be possible that the amount of PCSK9 bound is low and to overcome this it is essential to optimize the concentrations of proteins employed in this type of experiment. We also analyzed data regarding intra‐protein cross‐links (for both Hsp104fl and PCSK9). The data were filtered using the same parameters applied for the complex (Id‐score 15), resulting in pools of cross‐links formed exclusively within Hsp104 (ADH_DMTMM_ZL and DMTMM_ZL) and PCSK9 (DMTMM_ZL). We observed that nearly every region of Hsp104fl exhibits cross‐linking activity, with a predominance in the NBD1 and MD domains. Notably, for PCSK9, four cross‐links were identified involving residue 243 (243–206/210/212/238) (see Fig. S14). Our cross‐link data on Hsp104fl bound to an associated protein uncovered a robust approach to begin mapping how Hsp104 may interact with a particular substrate, which might be helpful in revealing general mechanisms of Hsp104 activity. It has to be emphasized that such use of XL‐MS data represents a novel approach to the analysis of protein complexes to understand how they interact.

## Discussion

**Fig. 6:**
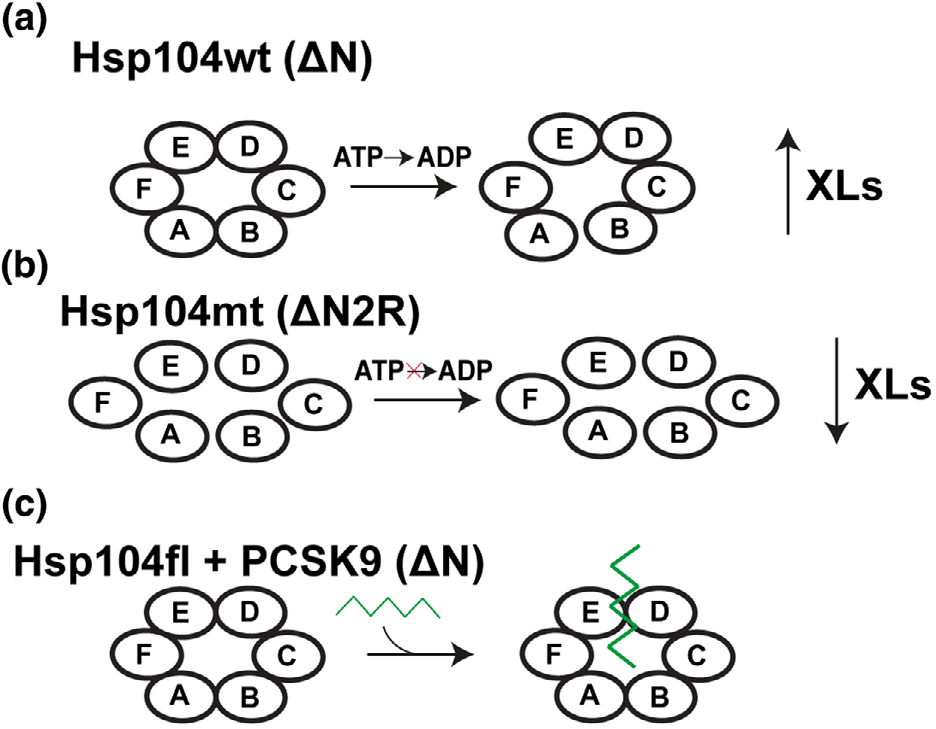
Asymmetry of Hsp104 detected by XL‐MS (cross‐linking mass spectrometry). (a) Wild‐type Hsp104 recognizes adenosine triphosphate (ATP) which mediates disaggregation. This feature has an impact on the yield of cross‐linking reaction. Moreover, Hsp104wt (wild‐type) structure is more condensed. (b) Genetically modified Hsp104mt (mutant) does not hydrolyze ATP. Compared with Hsp104wt, the efficiency and yield of cross‐linking reaction is reduced. These data suggest that the Hsp104mt may be more dynamic. (c) Hsp104fl (full length) interaction with PCSK9 yields the formation of several heterotypic cross‐links between the chaperone and the associated protein, which indicates that Hsp104fl can bind PCSK9 (proprotein convertase subtilisin/kexin type 9) to help refold it demonstrated by interactions to the central pore of Hsp104fl. Experiments were performed as one (*n* = 1) biologically independent replicate.

Hsp104 is a protein that plays a critical role in disaggregation of stress‐induced protein assemblies. In this study, we employed Hsp104wt and Hsp104mt that forms a hexamer to determine possible structural differences in these two complexes and to reveal Hsp104fl interactions with associated protein in order to elucidate which regions are involved in the formation of such construct. We determined structural differences between Hsp104wt and mt, revealing that Hsp104mt exhibits more expanded structure whereby this Hsp104 genetic variant is less susceptible to action of cross‐linking chemistry (Fig. 6a,b). As a consequence, for each chemistry we determined smaller number of cross‐links in Hsp104mt. Such structural asymmetry is especially discernible between A and F subunits—using our approach we determined two distinctive contacts (634/637–258) that only fit these two subunits in both X‐ray and cryo‐EM wild‐type Hsp104 hexamer. In addition, we revealed which Hsp104fl regions and PCSK9 amino acid residues are predominantly involved in the formation of Hsp104fl:PCSK9 complex. Two Hsp104fl regions (NBD1 and MD) and PCSK9 residue no: 243 mostly interact with each other, giving the disaggregation machinery that employs ATP to remove monomer units from protein assemblies. Our XL‐MS pipeline illustrates the ability to interpret changes in conformation of assemblies and how proteins may interact with a chaperone. Furthermore, we anticipate that new modeling approaches combined with XL‐MS will prove useful for improved modeling of assemblies.

### Different XL‐MS chemistries are consistent with structures

It is commonly known that Hsp104 not only disassembles toxic aggregates of protein but also provides its protective function in several amyloid‐related diseases. When expressed in yeast models, it promotes aggregation while its presence in animal cells may appear to regulate the clearance of amyloid assemblies [8]. In general, there are two main mechanisms of Hsp104 action. On the one hand, it uses its axial channel to disaggregate a substrate in an ATP‐driven manner, but on the other hand, it can also employ its NBD2 domain to capture amyloid client [20, 50]. Our approach to determine any conformational changes or asymmetries as well as residues and protein regions that are involved in interaction seems to be especially useful in such experiments where particular cross‐linking chemistry may have an impact on Hsp104 activity and/or structure. However, it has to be emphasized that an important limitation of this study is the need of prior models of the monomer to discriminate between intramolecular and intermolecular contacts to guide generation of the complex. We anticipate that machine learning based approaches for structure prediction of complexes combined with experimental cross‐links may help determine conformational heterogeneity of multi‐subunit complexes. Here, we employed two Hsp104 genetic variants (mt and wt) and structures (X‐ray and cryo‐EM) to reveal XL‐MS application in geometric and functional studies. We determined that all the cross‐links (generated using different chemistries) mapped onto both X‐ray and cryo‐EM structure fit perfectly to each of them—the only differences were caused by genetic variants of Hsp104 and thus Hsp104wt forms more links due to its more compact structure, while cross‐linking reaction of Hsp104mt leads to the formation of lower number of cross‐links. We revealed that in such experiments it is possible to employ any X‐ray or cryo‐EM structure without losing any important data. Nowadays, there is conviction that X‐ray crystallography and cryo‐EM are two competitive structural techniques with differences, such as sample preparation, accessibility and application; however, one should keep in mind that structural determination is just a preamble to something bigger, which is combining structural information with biological functions [51]. Hence, if it is possible to gain similar data in our XL‐MS approach using two different structures, there is no need to employ more expensive or less accessible technique.

### Visualization of putative substrates difficult using structural biology methods that rely on averaging but XL‐MS uncovers discrete binding

Cross‐linking reaction along with our novel analysis pipeline can be successfully employed to determine protein–protein interaction. Over the years, a lot of in‐depth studies have aimed to determine how Hsp104 interacts with protein assemblies/associated proteins [2, 10, 22, 49, 52, 53]. In general, X‐ray crystallography and cryo‐EM are commonly employed in protein structure determination. After refinement, such an approach provides a 3D model of protein and/or complex derived from many molecules observed in a crystal or a grid in X‐ray crystallography and cryo‐EM, respectively [54, 55]. In our experiments, we visualized cross‐links on X‐ray and cryo‐EM structures and revealed contacts/links consistent with asymmetric interactions observed in the staircase conformation of Hsp104. Furthermore, we use our XL‐MS information to interpret how an associated protein may interact with Hsp104fl (Fig. 6c). Having in mind that Hsp104 has an ability to alternate between two features (disaggregation and protection) [50], it is especially important to uncover each possible interaction of Hsp104 with other proteins, which helps to determine the exact model of Hsp104 activity.

## Conclusion

Our high‐resolution XL‐MS experiments combined with our analysis pipeline have uncovered nuances to the dynamics of Hsp104 forming the staircase conformation only previously visualized with cryo‐EM or X‐ray. Furthermore, our experiments have uncovered robust methods to interpret protein interactions with Hsp104 to gain insight into how substrates interact using XL‐MS alone. Using XL‐MS it is not only possible to confirm the protein structure but also to reveal its interaction with a substrate or with an associated protein, as in this case (Hsp104fl:PCSK9). Such an approach, based on the available cryo‐EM or X‐ray structures, combined with XL‐MS enables the detection of transient interactions (i.e., substrates) to begin to interpret mechanisms of binding/activity. Future experiments can be focused on a cross comparison of different proteins to identify general features of how proteins associate with Hsp104 ascertaining whether the interactions are consistent with a substrate or cofactor and mechanisms of translocation. It might be especially useful in the case of other members of AAA+ ATPase family, such as p97 and other molecular machines that maintain protein homeostasis to reveal not only how they interact with substrates or cofactors but also to reveal their mechanism of action [56]. Additionally, our approach may find application in revealing differences between the wild‐type and mutant proteins, which is of great importance in the case of mutants that can lead to a different phenotype [57] or mutants associated with disease. It also should be mentioned that the main goal of this research was to develop of a pipeline to interpret XL‐MS data in the context of homo‐oligomeric assemblies.

## Conflict of interest

The authors declare no conflict of interest.

### Peer review

The peer review history for this article is available at https://www.webofscience.com/api/gateway/wos/peer‐review/10.1002/2211‐5463.70007.

## Author contributions

KW, KM, AJ, and LAJ initiated the project. KM and KW characterized and purified the Hsp104 and Hsp104:PCSK9 complexes. KW performed the all cross‐linking experiments and analysis. KW and LAJ conceived and directed the research and wrote the manuscript. All authors contributed to the revisions of the manuscript.

## Supporting information


**Fig. S1.** WT Hsp104 and MT Hsp104 false discovery rate estimation.
**Fig. S2.** Mapping WT Hsp104 cross‐links onto ctHsp104 cryo‐EM structure.
**Fig. S3.** Mapping MT Hsp104 cross‐links onto ctHsp104 cryo‐EM structure.
**Fig. S4.** Mapping WT Hsp104 cross‐links onto ctHsp104 X‐ray structure.
**Fig. S5.** Mapping MT Hsp104 cross‐links onto ctHsp104 X‐ray structure.
**Fig. S6.** Hsp104 domain distribution of Hsp104wt and Hsp104mt cross‐links across chemistries that are consistent with the Hsp104 cryo‐EM structure.
**Fig. S7.** Evaluation of cross‐link mapping and structural match across FDR thresholds.
**Fig. S8.** Agreement between calculated cross‐link distances (in Å) from Hsp104wt between the Hsp104 X‐ray and cryo‐EM structures.
**Fig. S9.** Agreement between calculated cross‐link distances (in Å) from Hsp104mt between the Hsp104 X‐ray and cryo‐EM structures.
**Fig. S10.** Xlink‐peak MS2 spectra for cross‐links between 634 and 258 (ADH_DMTMM_ZL) and 637–258 (DMTMM_ZL) cross‐links from Hsp104wt consistent with A–F asymmetric geometry.
**Fig. S11.** Mapping geometry‐confirmed cross‐links WT ctHsp104 dataset onto the ctHsp104 cryo‐EM structure.
**Fig. S12.** Hsp104:PCSK9 false discovery rate estimation.
**Fig. S13.** Xlink‐peak MS2 spectra for cross‐links between 243–172, 243–505 and 243–813 (DMTMM_ZL) derived from PCSK9:Hsp104 complex.
**Fig. S14.** Hsp104fl and PCSK9 regions that form cross‐links.
**Table S1.** Sequences for Hsp104 (wt and variants) and PCSK9 used in the study.
**Table S2.** Molecular weight of Hsp104 constructs as determined by SEC of Hsp104 (wt and mut) and in complex with substrates.
**Table S3.** The number of cross‐links interpretable on structure and consistent with geometry of chemistry.
**Table S4.** Identified inter‐protein cross‐linked peptide pairs (Hsp104fl:PCSK9).

**Data S1.** Raw crosslinking mass spectrometry data.

## Data Availability

Raw XLMS data are available as source data 1. Raw mass spectrometry files for all cross‐link datasets are available on the MassIVE database under the following accession number MSV000090567 [https://doi.org/doi:10.25345/C5RB6W66D]. Any other data generated during and/or analyzed during the current study are available from the corresponding author on reasonable request.
