# Supplementary material for: Mapping Hsp104 interactions using cross‐linking mass spectrometry
Source: FEBS Open Bio. 2025 Feb 24;15(6):922–39. doi: 10.1002/2211-5463.70007 (PMC12127886; doi:10.1002/2211-5463.70007)
Supplement: Supplementary file 1 — Fig. S1. WT Hsp104 and MT Hsp104 false discovery rate estimation. Fig. S2. Mapping WT Hsp104 cross‐links onto ctHsp104 cryo‐EM structure. Fig. S3. Mapping MT Hsp104 cross‐links onto ctHsp104 cryo‐EM structure. Fig. S4. Mapping WT Hsp104 cross‐links onto ctHsp104 X‐ray structure. Fig. S5. Mapping MT Hsp104 cross‐links onto ctHsp104 X‐ray structure. Fig. S6. Hsp104 domain distribution of Hsp104wt and Hsp104mt cross‐links across chemistries that are consistent with the Hsp104 cryo‐EM structure. Fig. S7. Evaluation of cross‐link mapping and structural match across FDR thresholds. Fig. S8. Agreement between calculated cross‐link distances (in Å) from Hsp104wt between the Hsp104 X‐ray and cryo‐EM structures. Fig. S9. Agreement between calculated cross‐link distances (in Å) from Hsp104mt between the Hsp104 X‐ray and cryo‐EM structures. Fig. S10. Xlink‐peak MS2 spectra for cross‐links between 634 and 258 (ADH_DMTMM_ZL) and 637–258 (DMTMM_ZL) cross‐links from Hsp104wt consistent with A–F asymmetric geometry. Fig. S11. Mapping geometry‐confirmed cross‐links WT ctHsp104 dataset onto the ctHsp104 cryo‐EM structure. Fig. S12. Hsp104:PCSK9 false discovery rate estimation. Fig. S13. Xlink‐peak MS2 spectra for cross‐links between 243–172, 243–505 and 243–813 (DMTMM_ZL) derived from PCSK9:Hsp104 complex. Fig. S14. Hsp104fl and PCSK9 regions that form cross‐links. Table S1. Sequences for Hsp104 (wt and variants) and PCSK9 used in the study. Table S2. Molecular weight of Hsp104 constructs as determined by SEC of Hsp104 (wt and mut) and in complex with substrates. Table S3. The number of cross‐links interpretable on structure and consistent with geometry of chemistry. Table S4. Identified inter‐protein cross‐linked peptide pairs (Hsp104fl:PCSK9). [file FEB4-15-922-s002.docx]

**Supplementary Information**

**Mapping Hsp104 interactions using crosslinking mass spectrometry**

Kinga Westphal^1,2^, Karolina Michalska^3,4^, Andrzej Joachimiak^3,4,5^ and Lukasz A. Joachimiak^1,4^

^1^Center for Alzheimer’s and Neurodegenerative Diseases, Peter O’Donnell Jr. Brain Institute, University of Texas Southwestern Medical Center, Dallas, Texas 75390

^2^ Department of Medical Diagnostics, Centre for Advanced Materials and Technologies CEZAMAT, Warsaw University of Technology, 02-822 Warsaw, Poland

^3^Center for Structural Genomics of Infectious Diseases, Consortium for Advanced Science and Engineering, University of Chicago, Chicago, IL, 60667 USA

^4^Structural Biology Center, X-ray Science Division, Argonne National Laboratory, Lemont, IL, 60439 USA

^5^Department of Biochemistry and Molecular Biology, University of Chicago, Chicago, IL, 60367 USA

**Source data 1. Raw XL-MS files for WT Hsp104, mutant Hsp104 and full length Hsp104 in complex with PCSK9 substrate.**

**Supplementary Tables**

| **Hsp104**  **Thermochaetoides thermophila**  **(full length 1-926)**  **(Hsp104fl)** | MNSKMEFTDRAKKALEDAMALAEQYQHLQLQPVHLAVALLDPTPDPSKDQSIAPGTTSTLFRQVVERAHGDAQAFDRALKKKLVRLPSQDPPPDQVSMSAGCSNVLRKANELQKVQKDSYIAVDHLIAALAEDHAIQEALKEANIPKPKLIQDAIQAIRGNKRVDSRNADTEQENENLSKFCIDMTAMAREGKIDPVIGREEEIRRVIRILSRRTKNNPVLIGEPGVGKTTIVEGLAQRIVNADVPDNLAACKLLSLDVGALVAGSKYRGEFEERMKGVLKEIQESKETIILFVDEIHLLMGAGSSGEGGMDAANLLKPMLARGQLHCIGATTLAEYRKYIEKDAAFERRFQQVLVKEPSISETISILRGLKEKYEVHHGVNIADAAIVAAANLAARYLTSRRLPDSAVDLIDEAAAAVRVARESQPEIIDSLERRLRQLKIEIHALSREKDEASKARLAQAKQDAQNVEEELRPLREKY  ERERQRGKAIQEAKMKLEALRVKAEDASRMGDHSRAADLQYYAIPEQEAIIKRLEAEKAAADAALNANGADVGGSMITDVVGPDQINEIVARWTGIPVTRLKTSEKEKLLHMEQALSKIVVGQKEAVQSVSNAIRLQRSGLSNPNQPPSFLFCGPSGTGKTLLTKALAEFLFDDPKSMIRFDMSEYQERHSLSRMIGAPPGYVGHDAGGQLTEALRRRPFSILLFDEVEKAAKEVLTVLLQLMDDGRITDGQGRVVDAKNCIVVMTSNLGAEYLSRANNGKDGKIDPTTRELVMNTLRNYFLPEFLNRISSVVIFNRLTRREIRKIVDLRIAEIQKRLTDNDRNVIIKVSEEAKDKLGAQGYSPVYGARPLQRLLEKEVLNRLAILILRGQIREGEVAHVELVDGKVQVLPNHPDSEPEDVDVDMDSDDAVDEVAPDSMDEDIYND |
| --- | --- |
| **Hsp104 ΔN**  **Calcarisporiella thermophila (153-883)**  **(Hsp104wt)** | AEEAYEALSKYCIDLTELAASGKLDPVIGRDEIISRVIRVLSRRTKNNPCLVGEPGVGKTAIAEGLANRIVKGDIPSSLQKKVYSLDIGSLLAGAKYRGEFEERLKAVLKELKEAQAIVFIDEIHTVLGAGKSEGAIDAANLLKPMLARGELRCIGATTLTEYRQYVEKDPAFERRFQLVMVEEPSVTDTISILRGLKERYETHHGVRIADAAIVAAAQLAARYITQRFMPDKAIDLIDEACANTRVQLDSQPEAIDKLERRHLQLEVEATALEKEKDAASKQRLQEVRAEMARIQEELRPLKMKYESEKGRLDEIRNLSQRLDELKAKAEDAERRYDLARAADIRYYAIPDLEKRLAQLQAEKSQADAERADGLLAEVVGPDQIMEVVSRWTGIPVSNLQRSEKEKLLHMEEYMKQHVVGQDEAIKAICDAIRLSRTGLQNRNRPLASFLFLGPTGCGKTLCVKELAAFLFNDPGAIVRIDMSEYMEKHAVSRLVGAPPGYIGHDEGGQLTEAVRRRPYTVVLFDEMEKAHKDVSNLLLQILDDGHCTDSKGRRVDFKNTIIVMTSNLGADLFELDEGDKVSQATKNAVLATARRHFANEFINRIDELIVFNRLTPSNIRKIVDVRLKEVQERLDEKQITLDVDDKAKDLLAQQGFDPVYGARPLNRLIQHALLTQLSRLLLDGGVRPGEIAKVTVDQEGEIIVIRNHGIESPAPWADEDMVEDEDMEI |
| **Hsp104 ΔN R328M/R757M**  **Calcarisporiella thermophila (153-883) (Hsp104mt)**  Mutations are in bold and underlined. | AEEAYEALSKYCIDLTELAASGKLDPVIGRDEIISRVIRVLSRRTKNNPCLVGEPGVGKTAIAEGLANRIVKGDIPSSLQKKVYSLDIGSLLAGAKYRGEFEERLKAVLKELKEAQAIVFIDEIHTVLGAGKSEGAIDAANLLKPMLARGELRCIGATTLTEYRQYVEKDPAFE**R**RFQLVMVEEPSVTDTISILRGLKERYETHHGVRIADAAIVAAAQLAARYITQRFMPDKAIDLIDEACANTRVQLDSQPEAIDKLERRHLQLEVEATALEKEKDAASKQRLQEVRAEMARIQEELRPLKMKYESEKGRLDEIRNLSQRLDELKAKAEDAERRYDLARAADIRYYAIPDLEKRLAQLQAEKSQADAERADGLLAEVVGPDQIMEVVSRWTGIPVSNLQRSEKEKLLHMEEYMKQHVVGQDEAIKAICDAIRLSRTGLQNRNRPLASFLFLGPTGCGKTLCVKELAAFLFNDPGAIVRIDMSEYMEKHAVSRLVGAPPGYIGHDEGGQLTEAVRRRPYTVVLFDEMEKAHKDVSNLLLQILDDGHCTDSKGRRVDFKNTIIVMTSNLGADLFELDEGDKVSQATKNAVLATARRHFANEFIN**R**IDELIVFNRLTPSNIRKIVDVRLKEVQERLDEKQITLDVDDKAKDLLAQQGFDPVYGARPLNRLIQHALLTQLSRLLLDGGVRPGEIAKVTVDQEGEIIVIRNHGIESPAPWADEDMVEDEDMEI |
| **PCSK9 ΔN (153-692)**  **(PCSK9)** | SIPWNLERITPPRYRADEYQPPDGGSLVEVYLLDTSIQSDHREIEGRVMVTDFENVPEEDGTRFHRQASKCDSHGTHLAGVVSGRDAGVAKGASMRSLRVLNCQGKGTVSGTLIGLEFIRKSQLVQPVGPLVVLLPLAGGYSRVLNAACQRLARAGVVLVTAAGNFRDDACLYSPASAPEVITVGATNAQDQPVTLGTLGTNFGRCVDLFAPGEDIIGASSDCSTCFVSQSGTSQAAAHVAGIAAMMLSAEPELTLAELRQRLIHFSAKDVINEAWFPEDQRVLTPNLVAALPPSTHGAGWQLFCRTVWSAHSGPTRMATAVARCAPDEELLSCSSFSRSGKRRGERMEAQGGKLVCRAHNAFGGEGVYAIARCCLLPQANCSVHTAPPAEASMGTRVHCHQQGHVLTGCSSHWEVEDLGTHKPPVLRPRGQPNQCVGHREASIHASCCHAPGLECKVKEHGIPAPQEQVTVACEEGWTLTGCSALPGTSHVLGAYAVDNTCVVRSRDVSTTGSTSEGAVTAVAICCRSRHLAQASQELQ |

**Supplementary Table 1.** Hsp104 (wt and variants) and PCSK9 sequences used in the study.

| Protein | Protein construct (AA range) | SEC M_r_ (kDa) | MW monomer | Expected MW of complex |
| --- | --- | --- | --- | --- |
| CtHsp104ΔN (Hsp104wt) | 153-883 | 522.0 | 81.8 | 490.8 (hexamer) |
| CtHsp104ΔN2R (Hsp104mt) (R328M/R757M) | 153-883 | 513.2 | 81.7 | 490.2 (hexamer) |
| TtHsp104:PCK9 ΔN  (Hsp104fl:PCSK9) | 1-926 (Hsp104fl), 153-592 (PCSK9) | 673.0 | 102.8_Hsp104_  57.3_PCKS9_ | 674.1  (hexamer+substrate) |

**Supplementary Table 2.** Molecular weight of Hsp104 constructs as determined by SEC of Hsp104 (wt and mut) and in complex with substrates.

**Supplementary Figures and Legends**


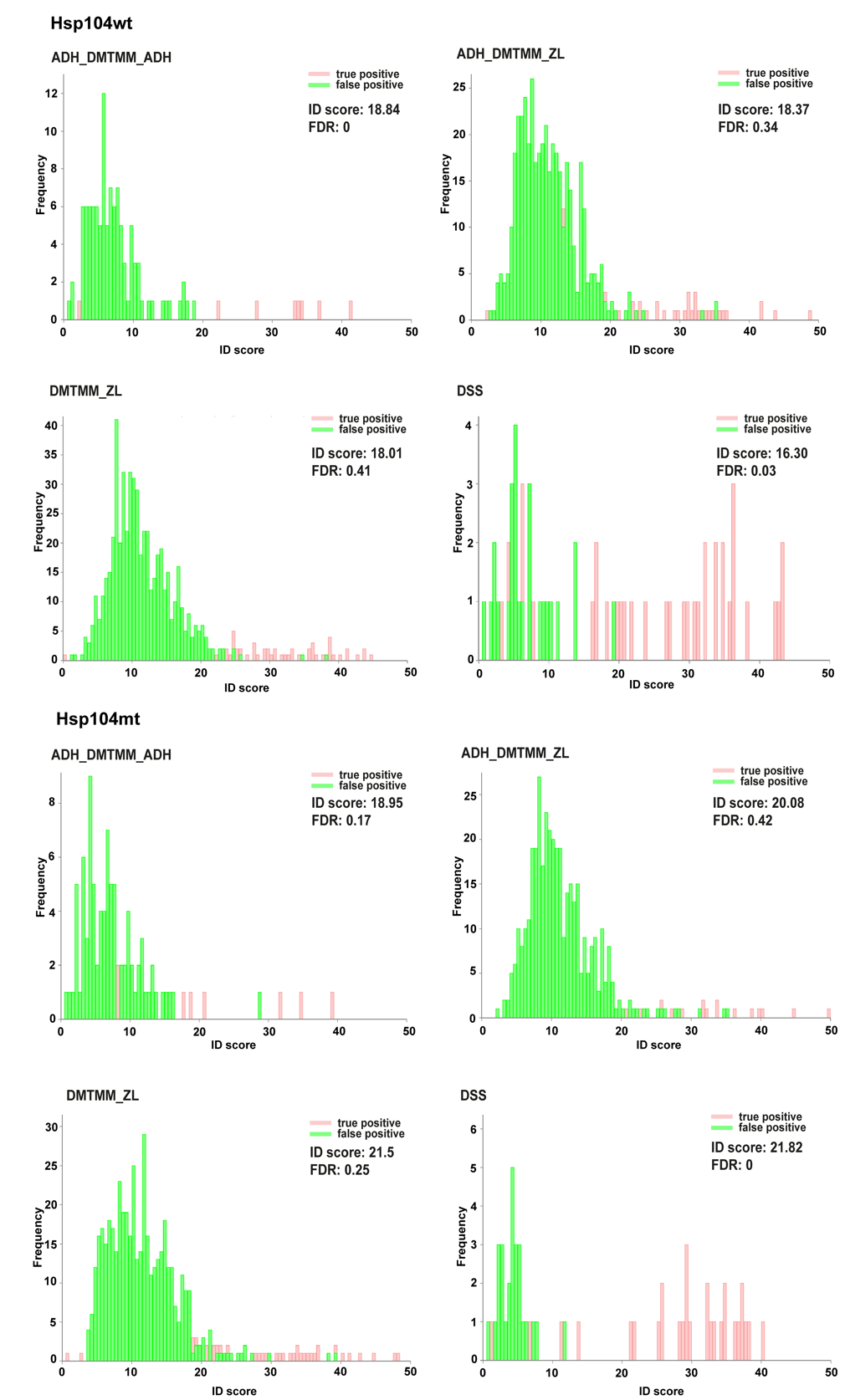


**Supplementary Figure 1. Wt Hsp104 and mt Hsp104 false discovery rate estimation.** Representative true positive (red) and false positive (green) distribution plots separated by Id-score to calculate false discovery rates (FDRs) for each XL-MS dataset. False positives (FPs) are defined as hits to the decoy sequences. True positives (TPs) are defined as hits to the true sequence. FDRs are calculated by calculating the fraction of FPs divided by the sum of FP+TP at a specific Id-score.

**
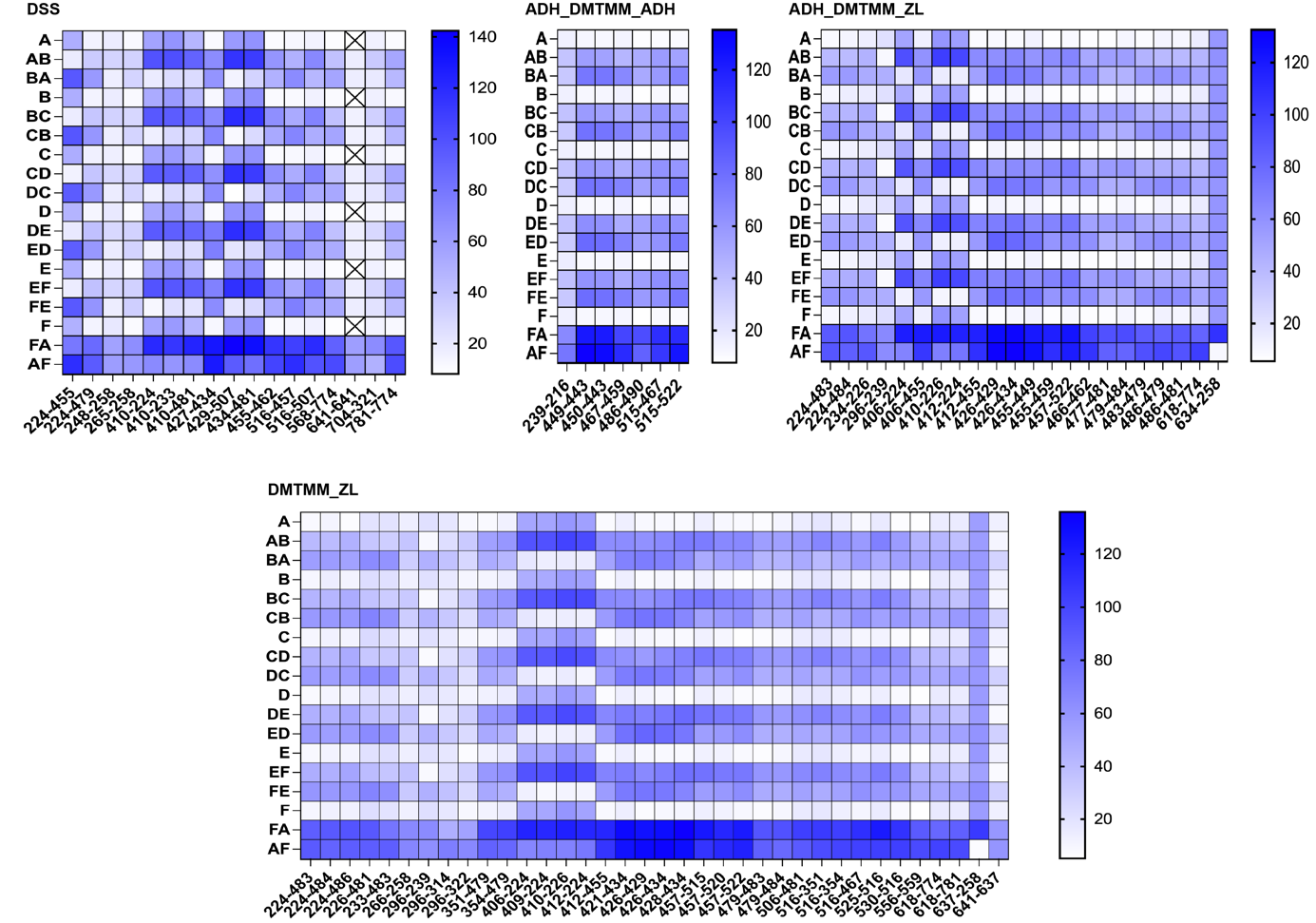
**

**Supplementary Figure 2. Mapping WT Hsp104 crosslinks onto ctHsp104 cryo-EM structure.** Heat maps represent crosslinks formed within one unit or between two neighboring units in Hsp104 wt cryo-EM structure, using DSS, ADH_DMTMM and DMTMM. Coloring represents the distance (Å) between two linked residues, where the greater the distance, the darker the color. Heatmap plots were generated using GraphPad Prism v9.4.1 (https://www.graphpad.com/).

**
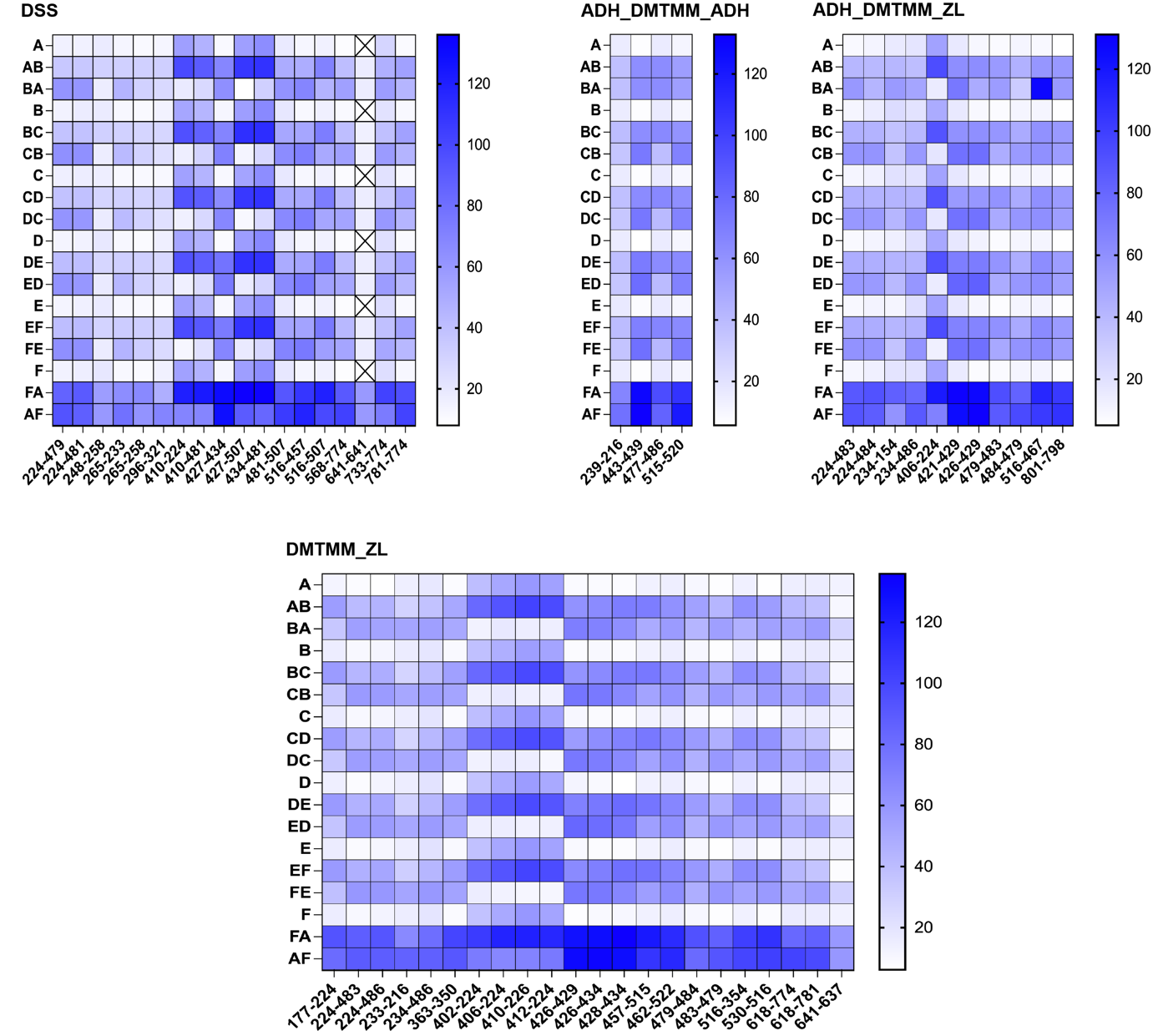
**

**Supplementary Figure 3. Mapping MT Hsp104 crosslinks onto ctHsp104 cryo-EM structure.** Heat maps represent crosslinks formed within one unit or between two neighboring units in Hsp104mt cryo-EM structure, using DSS, ADH_DMTMM and DMTMM. Coloring represents the distance (Å) between two linked residues, where the greater the distance, the darker the color. Heatmap plots were generated using GraphPad Prism v9.4.1 (https://www.graphpad.com/).

**
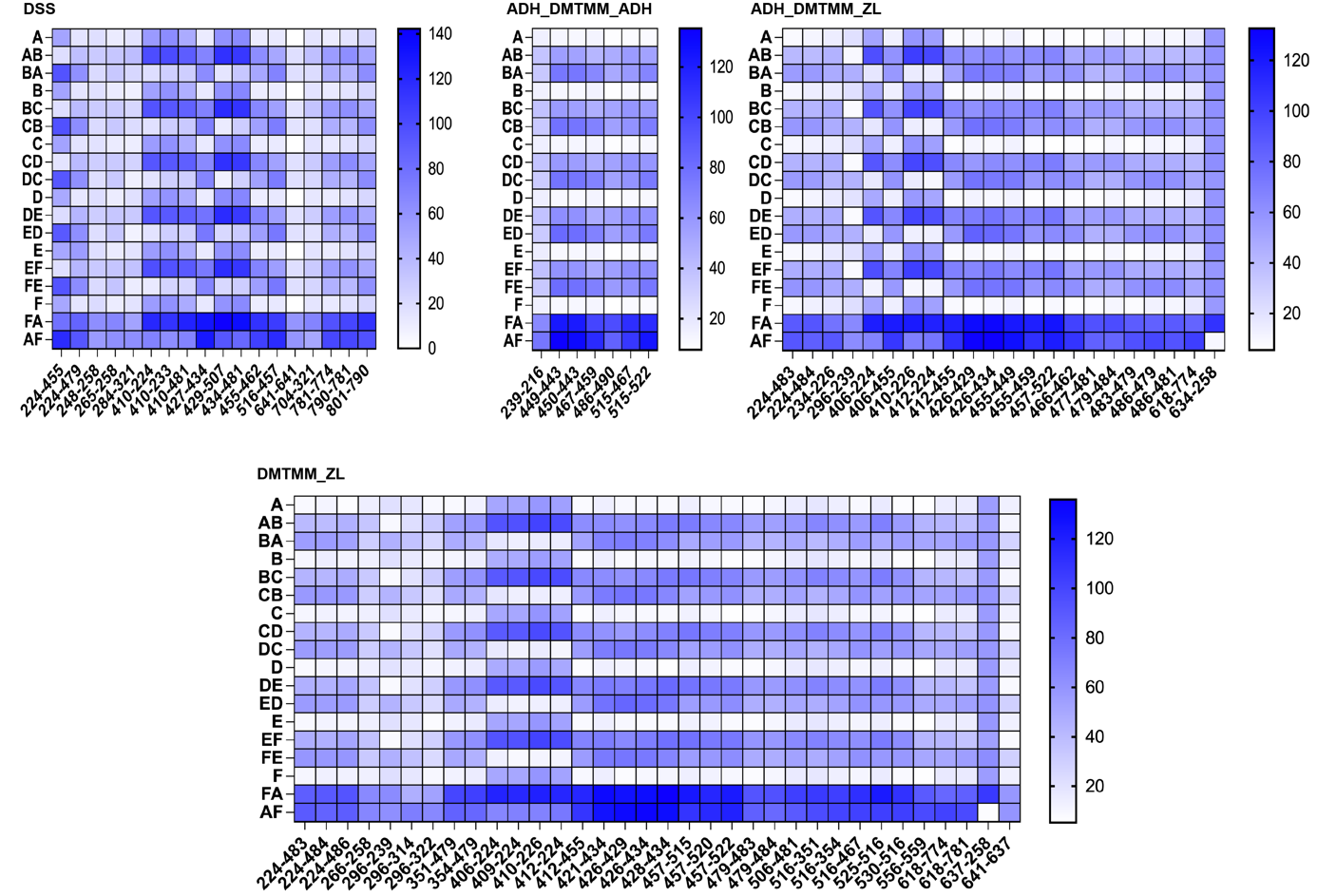
**

**Supplementary Figure 4. Mapping WT Hsp104 crosslinks onto ctHsp104 X-ray structure.** Heat maps represent crosslinks formed within one unit or between two neighboring units in Hsp104 wt X-ray structure, using DSS, ADH_DMTMM and DMTMM. Coloring represents the distance (Å) between two linked residues, where the greater the distance, the darker the color. Heatmap plots were generated using GraphPad Prism v9.4.1 (https://www.graphpad.com/).


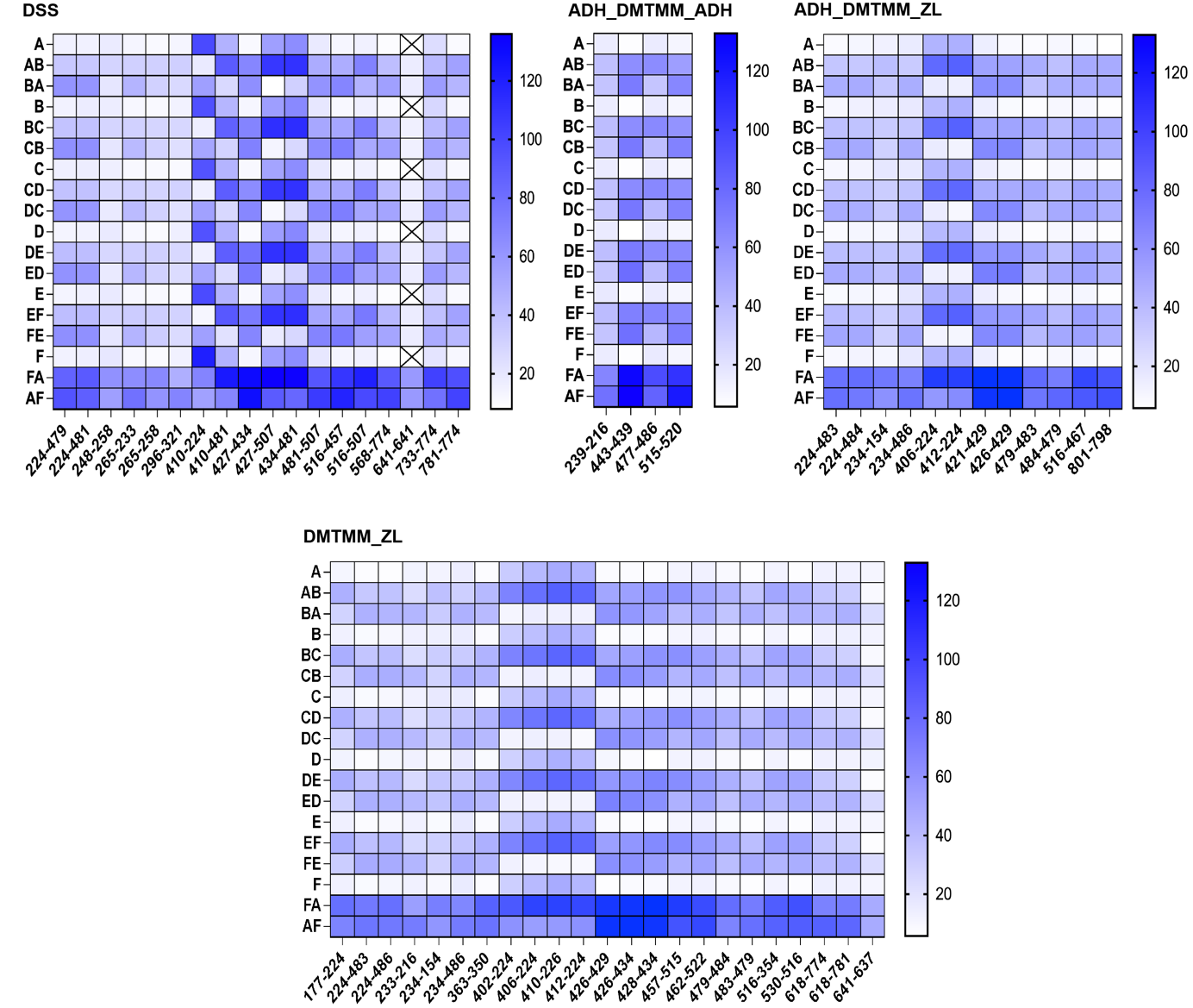


**Supplementary Figure 5. Mapping MT Hsp104 crosslinks onto ctHsp104 X-ray structure.** Heat maps represent cross-links formed within one unit or between two neighboring units in Hsp104mt cryo-EM structure, using DSS, ADH_DMTMM and DMTMM. Coloring represents the distance (Å) between two linked residues, where the greater the distance, the darker the color. Heatmap plots were generated using GraphPad Prism v9.4.1 (https://www.graphpad.com/).

**
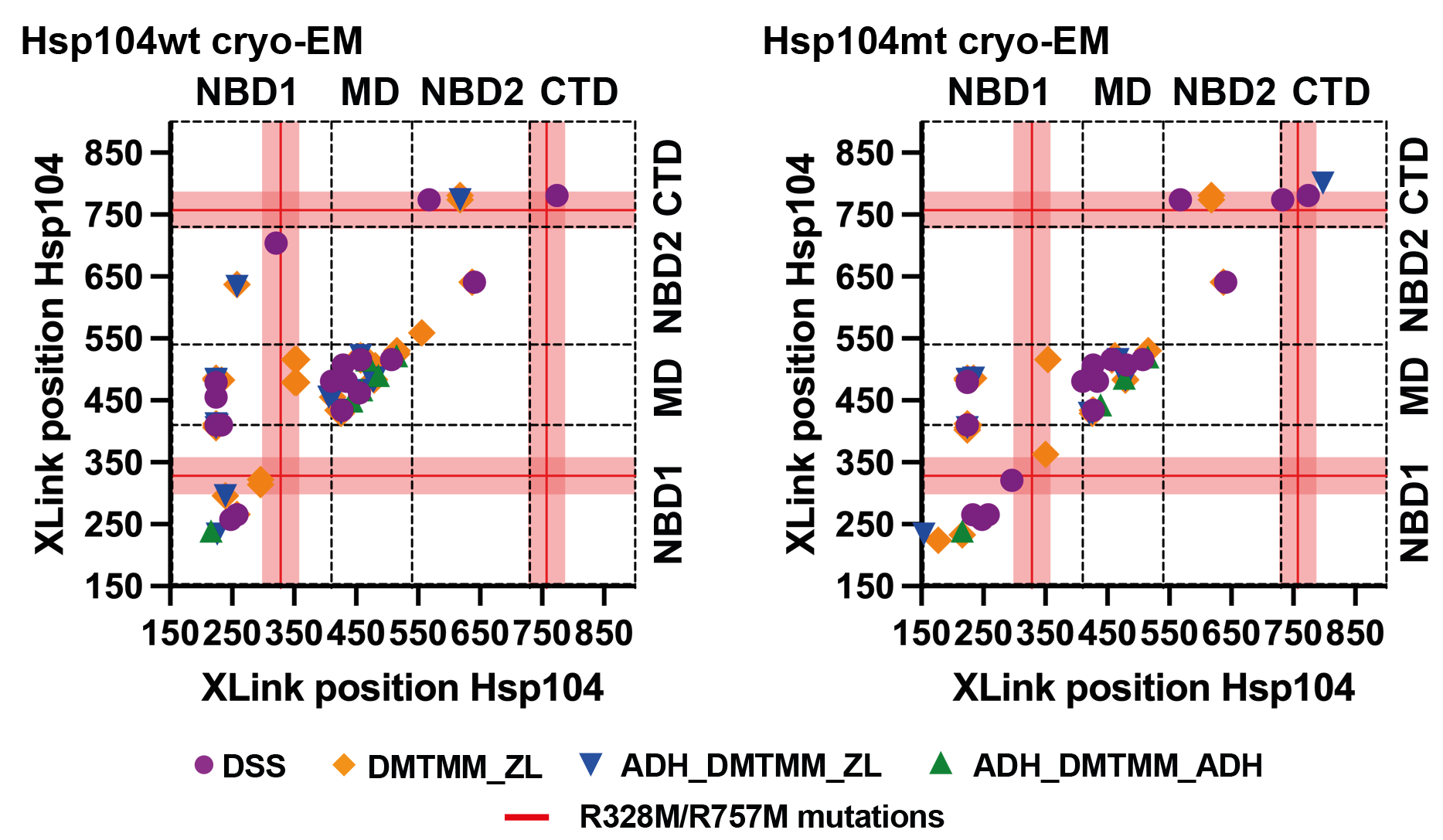
**

**Supplementary Figure 6. Hsp104 domain distribution of Hsp104wt and Hsp104mt crosslinks across chemistries that are consistent with the Hsp104 cryo-EM structure.**

Crosslinked pairs plotted to show connectivity of contacts within and across domains from the Hsp104wt/mt XL-MS datasets showing only contacts that were consistent with the experimental cryo-EM Hsp104 structure. The largest number of crosslinks was formed within NBD1 and MD domains. Only minor differences can be detected between Hsp104wt and Hsp104mt (Hsp104mt forms fewer cross-links). In regions proximal to the mutated residues in Hsp104mt, less cross-links were detected in Hsp104mt (7 cross-links) compared to Hsp104wt (10 cross-links) as defined as 328±30 and 757±30 (red lines indicate sites of mutation and colored area includes region considered as hits). Cross-links are colored by chemistry and are shown in purple, orange, blue and green for DSS, DMTMM_ZL, ADH_DMTMM_ZL and ADH_DMTMM_ADH, respectively. Domains are separated by dashed lines.


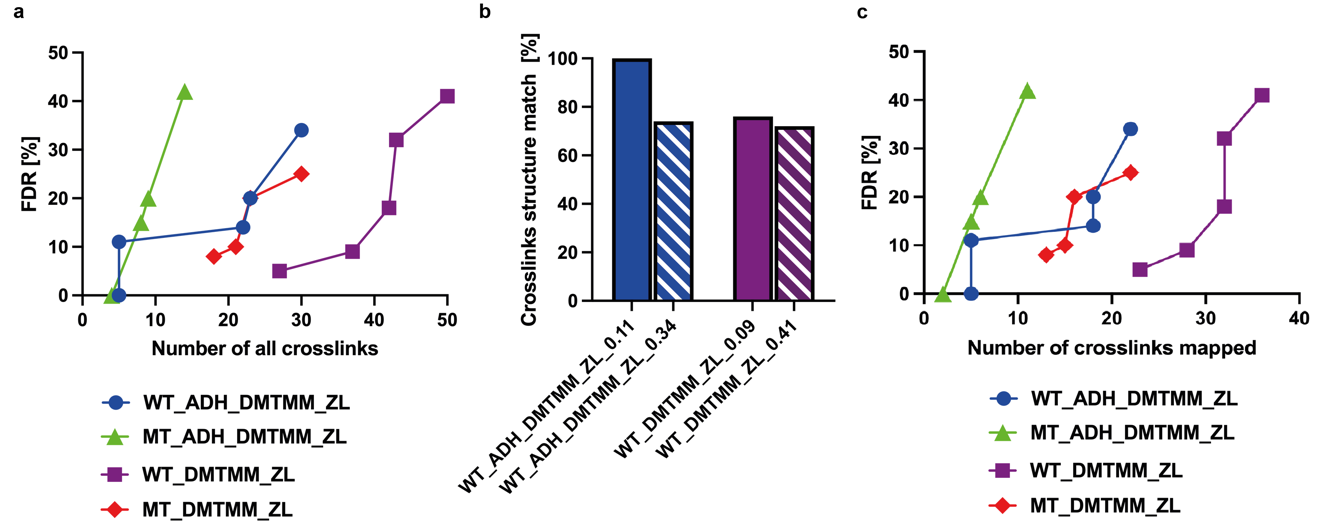


**Supplementary Figure 7. Evaluation of crosslink mapping and structural match across FDR thresholds. a.** Number crosslinks for Hsp104wt and Hsp104mt across FDR values for ADH_DMTMM_ZL and DMTMM_ZL datasets. **b.** Percentage of crosslinks consistent with geometries for experimental Hsp104 cryo-EM structure obtained for Hsp104wt using ADH_DMTMM_ZL and DMTMM_ZL on the Hsp104 cryo-EM structure for FDR values of 11% and 34% (ADH_DMTMM_ZL) and 9% and 42% (DMTMM_ZL). **c.** Number of crosslinks consistent with experimental Hsp104 structure for Hsp104wt and Hsp104mt across FDR values using ADH_DMTMM_ZL and DMTMM_ZL.


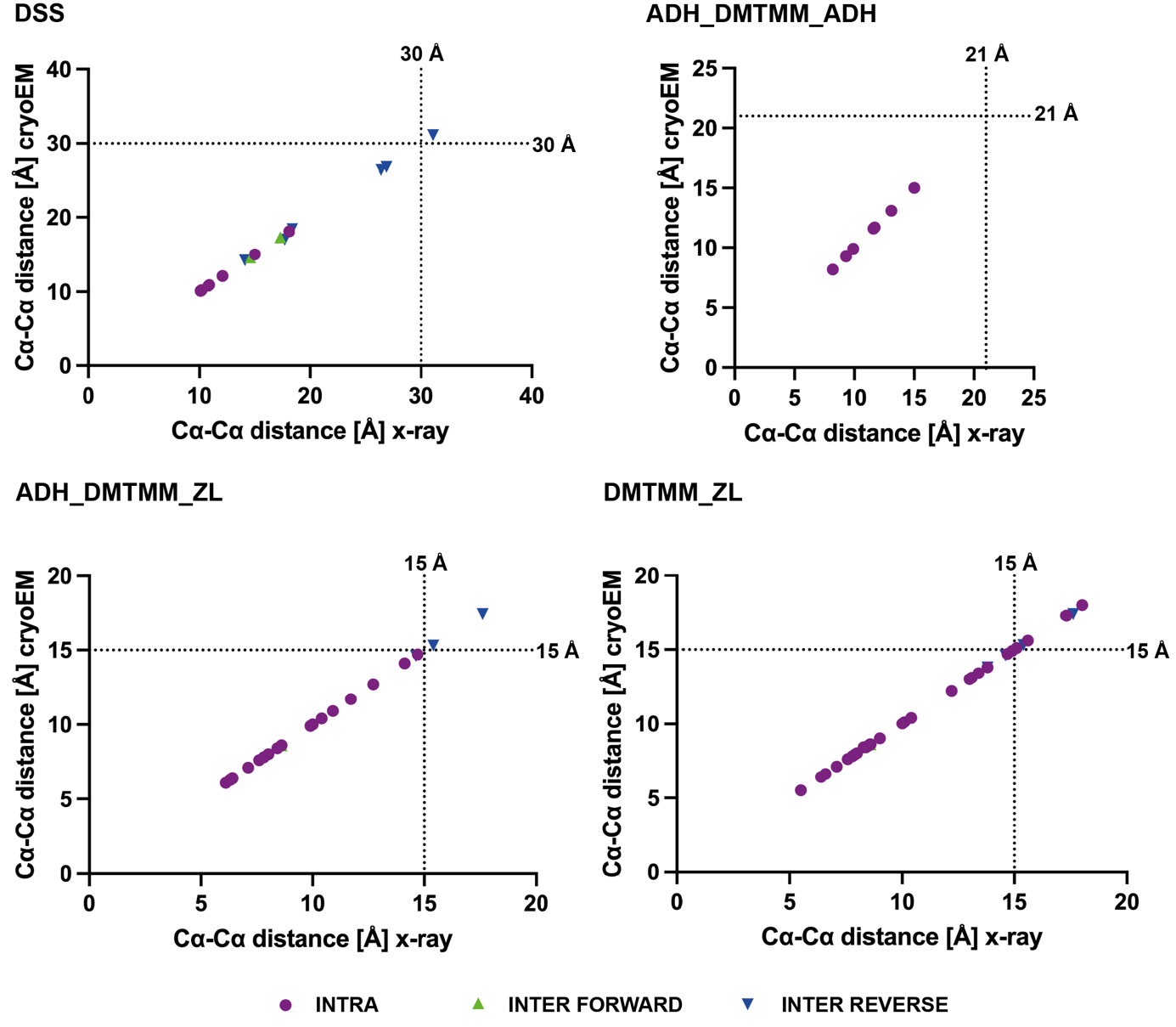


**Supplementary Figure 8. Agreement between calculated crosslink distances (in Å) from Hsp104wt between the Hsp104 x-ray and cryo-EM structures.** Each cross-link derived from the datasets derived from the Hsp104wt complex is mapped onto x-ray and cryo-EM structure and the distances compared directory. This approach revealed that the geometries of the Hsp104 x-ray and cryo-EM structures are similar. The INTRA, INTER FORWARD and INTER REVERSE crosslink geometries are presented as purple dots, green triangles and blue triangles, respectively. The dotted lines indicate the distance threshold (in Å) for satisfying each chemistry.


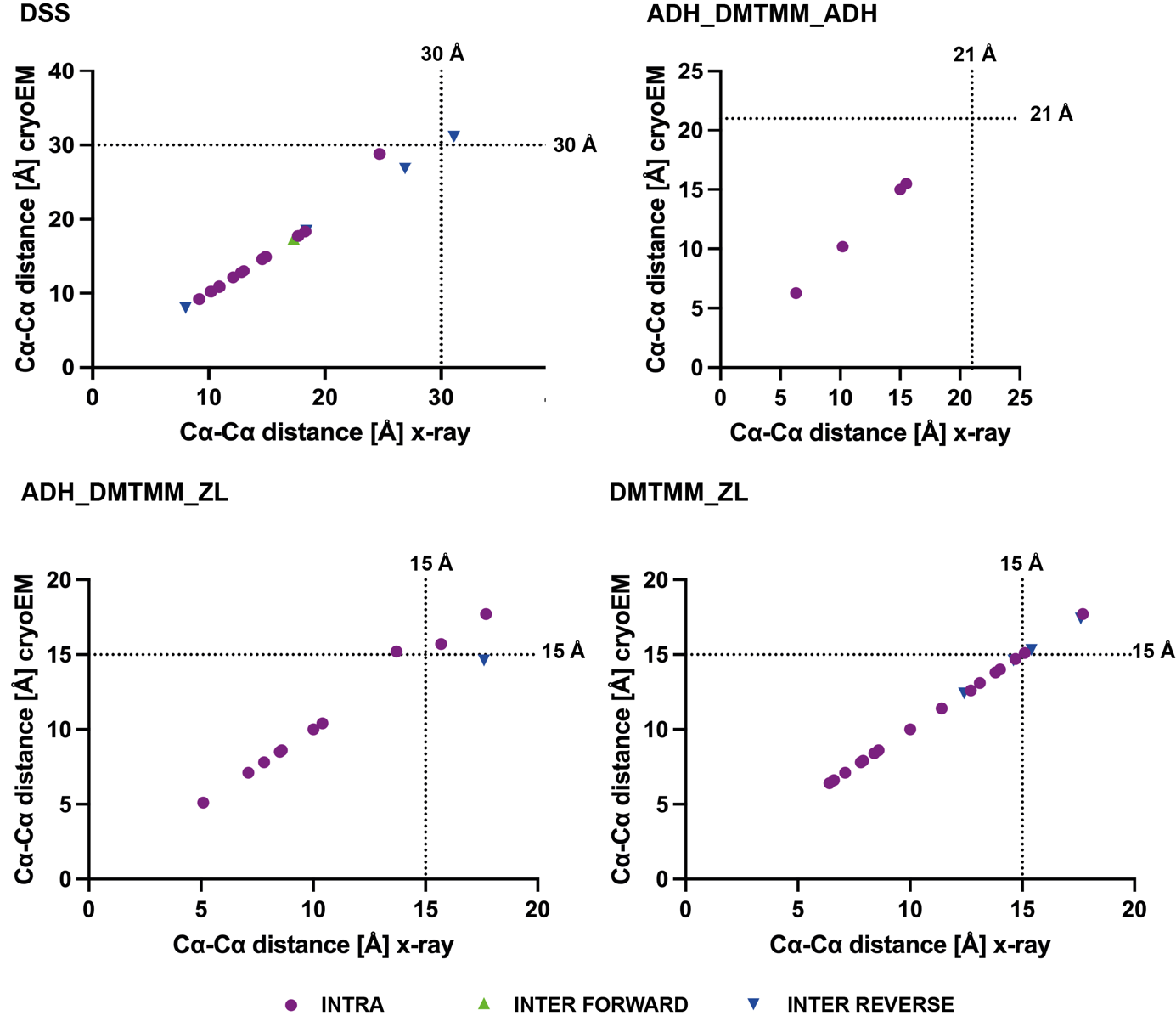


**Supplementary Figure 9. Agreement between calculated crosslink distances (in Å) from Hsp104mt between the Hsp104 X-ray and cryo-EM structures.** Each cross-link derived from the datasets derived from the Hsp104mt complex is mapped onto x-ray and cryo-EM structure and the distances compared directory. This approach revealed that the geometries of the Hsp104 x-ray and cryo-EM structures are similar. The INTRA, INTER FORWARD and INTER REVERSE crosslink geometries are presented as purple dots, green triangles and blue triangles, respectively. The dotted lines indicate the distance threshold (in Å) for satisfying each chemistry.

**
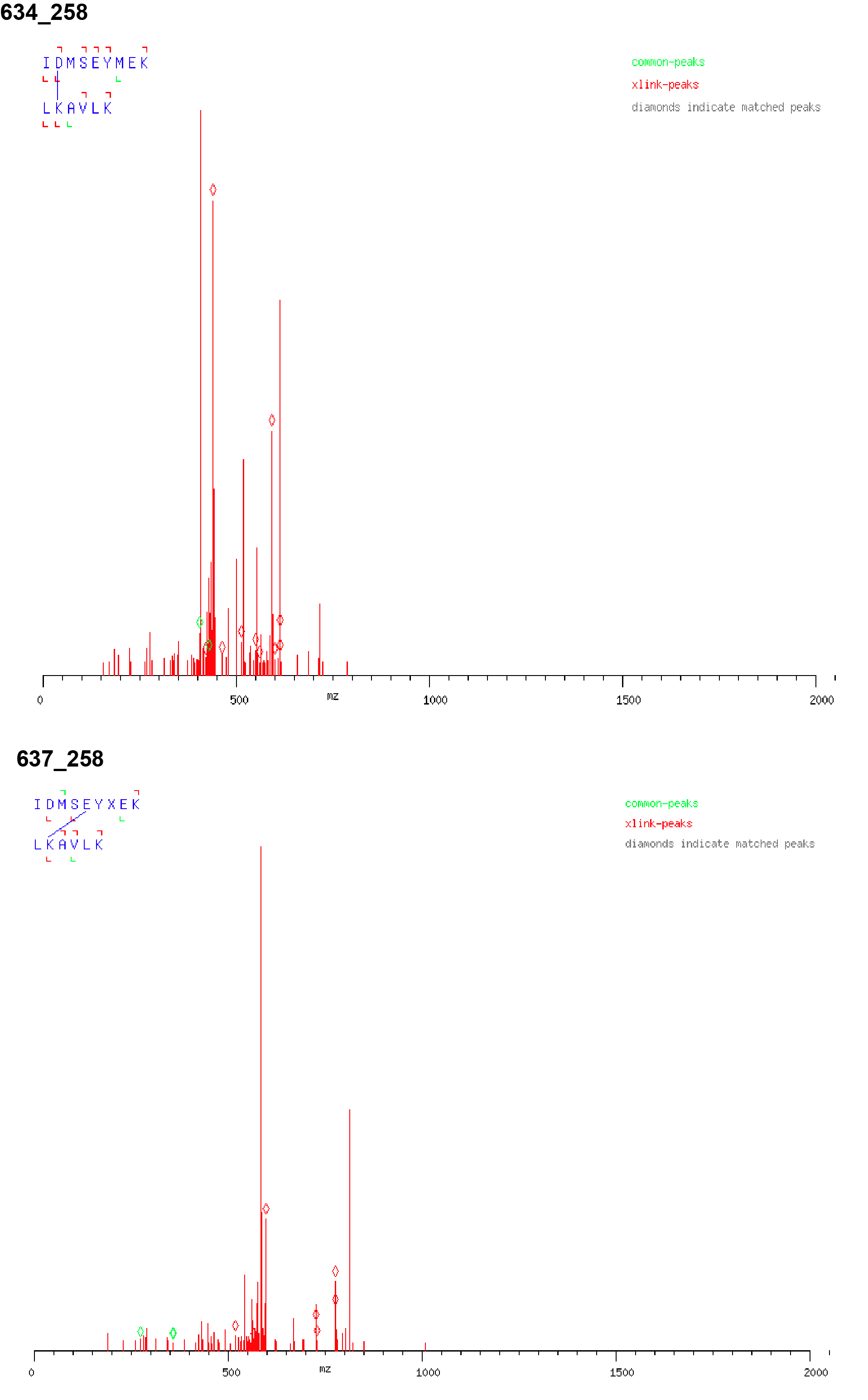
**

**Supplementary Figure 10. Xlink-peak MS2 spectra for crosslinks between 634-258 (ADH_DMTMM_ZL) and 637-258 (DMTMM_ZL) crosslinks from Hsp104wt consistent with A-F asymmetric geometry.**


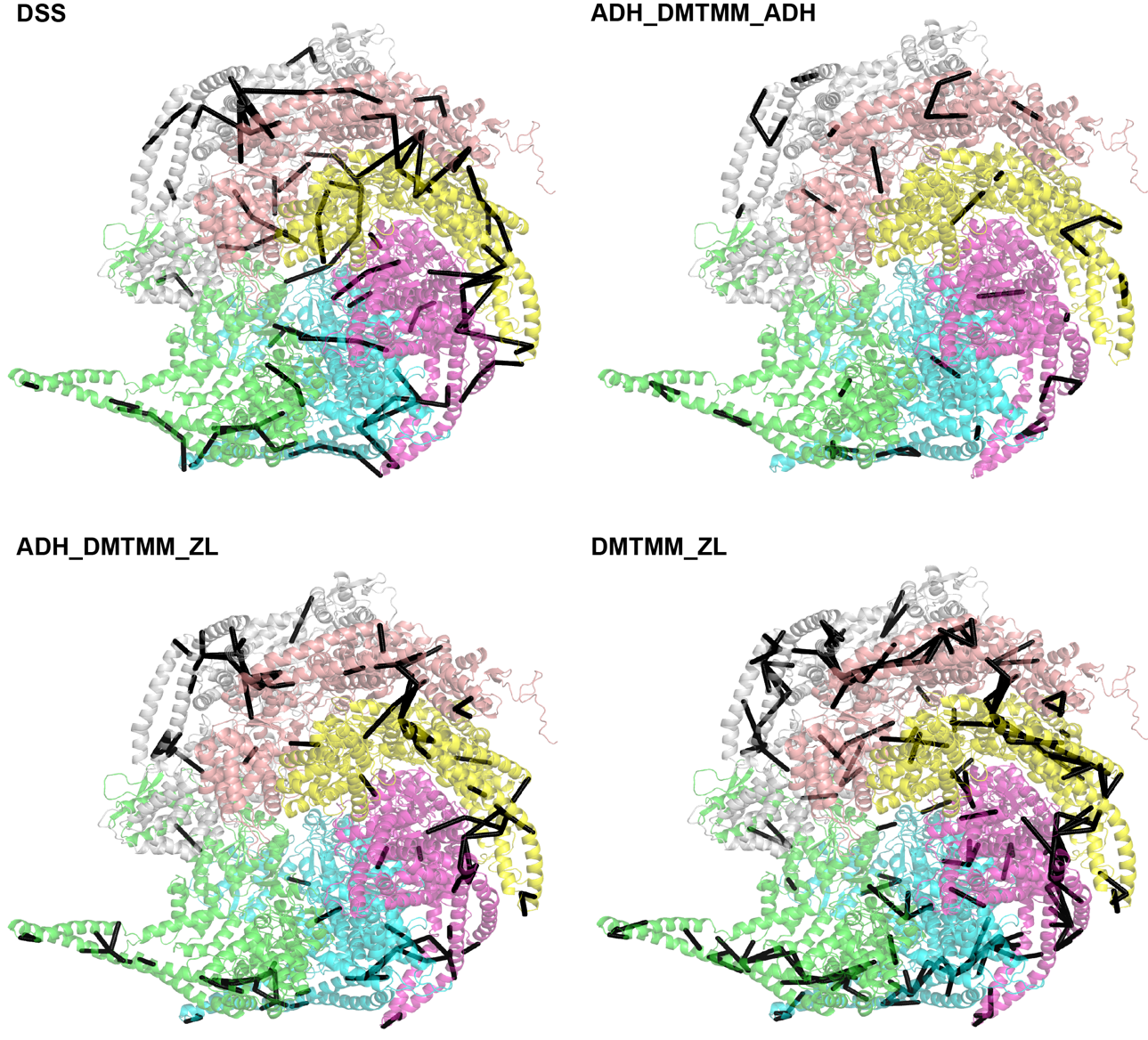


**Supplementary Figure 11. Mapping geometry-confirmed crosslinks WT ctHsp104 dataset onto the ctHsp104 cryo-EM structure.** Representative crosslinks mapped onto Hsp104wt cryo-EM structure. All crosslinks identified to be below the distance threshold for each chemistry (i.e. Supplementary Figures 7 and 8) were mapped onto cryo-EM structure to reveal the network of XL-MS interactions geometry and identify regions accessible to the crosslinkers. Since dataset obtained for both structure (cryo-EM and X-ray) and genetic variants (wild type and mutant) are similar, only mapping onto ctHsp104 cryo-EM structure is included.

|  | **Total Crosslinks** | **Crosslinks interpretable on structure** | **Crosslinks that satisfy distance cut-off** | **Fraction of crosslinks that satisfy distance cut-off [%]** | | |
| --- | --- | --- | --- | --- | --- | --- |
| **Hsp104wt (cryo-EM and X-ray)** | | | | | | |
| **DSS** | 23 | 18 (17 for cryo-EM) | 17  (16 for cryo-EM) | | 94 | |
| **ADH_DMTMM_ADH** | 7 | 7 | 7 | | 100 | |
| **ADH_DMTMM_ZL** | 30 | 22 | 20 | | 91 | |
| **DMTMM_ZL** | 50 | 34  36 (for cryo-EM) | 28  (30 for cryo-EM) | | 82  (83 for cryo-EM) | |
| **Hsp104mt (cryo-EM and X-ray)** | | | | | | |
| **DSS** | 19 | 18 | 17 | | | 94 |
| **ADH_DMTMM_ADH** | 4 | 4 | 4 | | | 100 |
| **ADH_DMTMM_ZL** | 14 | 12  (11 for  cryo-EM) | 9  (8 for cryo-EM) | | | 75  (73 for  cryo-EM) |
| **DMTMM_ZL** | 30 | 23  (22 for cryo-EM) | 19  (18 for cryo-EM) | | | 83  (82 for cryo-EM) |

**Supplementary Table 3.** The number of crosslinks interpretable on structure and consistent with geometry of chemistry.

| **Hsp104fl:PCSK9** | | | |
| --- | --- | --- | --- |
| **ADH_DMTMM_ZL** | | **DMTMM_ZL** | |
| Hsp104 | PCSK9 | Hsp104 | PCSK9 |
| 162 | 512 | 108 | 212 |
| 180 | 669 | 114 | 498 |
| 609 | 501 | 172 | 243 |
|  |  | 277 | 238 |
|  |  | 452 | 243 |
|  |  | 483 | 506 |
|  |  | 505 | 243 |
|  |  | 598 | 660 |
|  |  | 813 | 243 |

**Supplementary Table 4. Identified inter-protein cross-linked peptide pairs (Hsp104fl:PCSK9).** Table displays the identified inter-protein cross-linked peptide pairs, highlighting their linking sites.


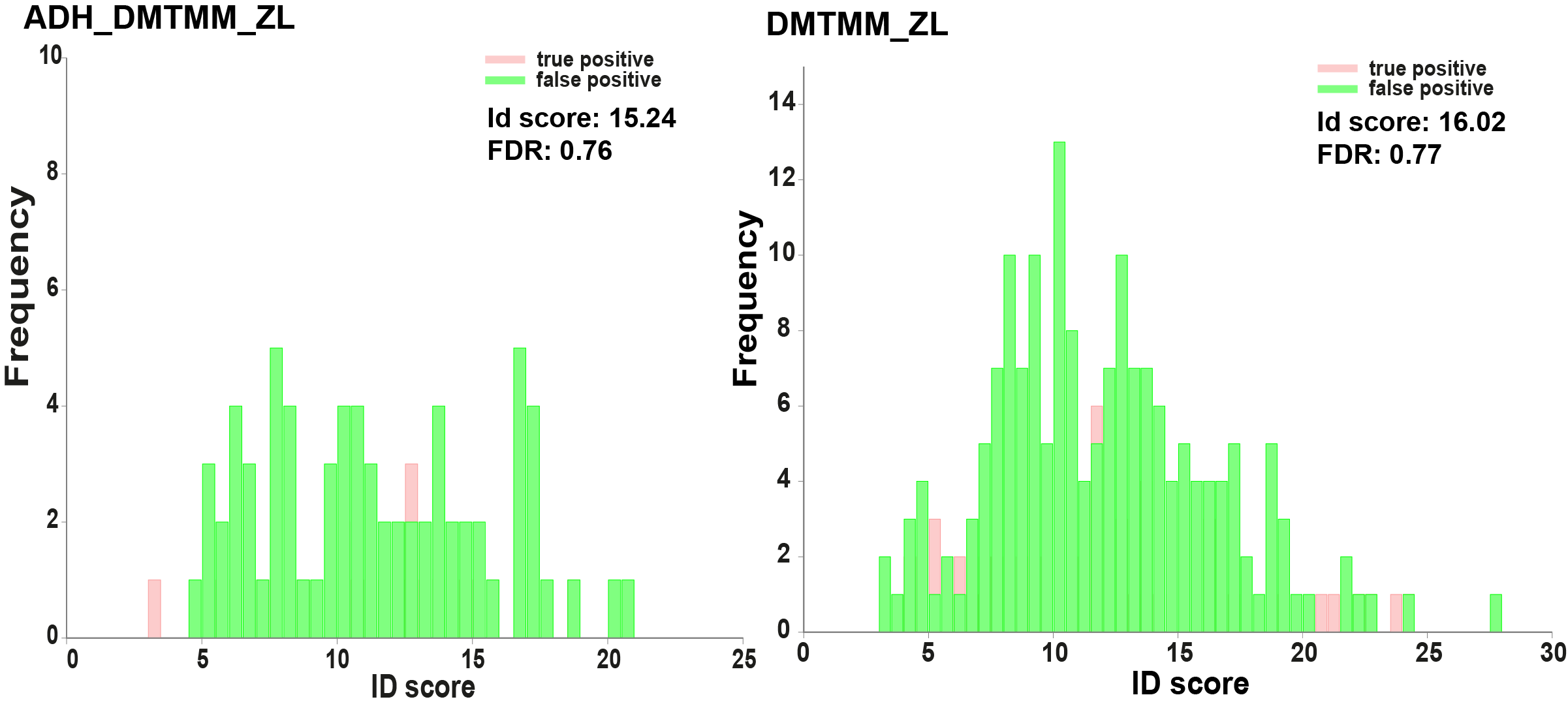


**Supplementary Figure 12. Hsp104:PCSK9 false discovery rate estimation.**

Representative true positive (red) and false positive (green) distribution plots separated by Id-score to calculate false discovery rates (FDRs) for each XL-MS dataset. False positives (FPs) are defined as hits to the decoy sequences. True positives (TPs) are defined as hits to the true sequence. FDRs are calculated by calculating the fraction of FPs divided by the sum of FP+TP at a specific Id-score.


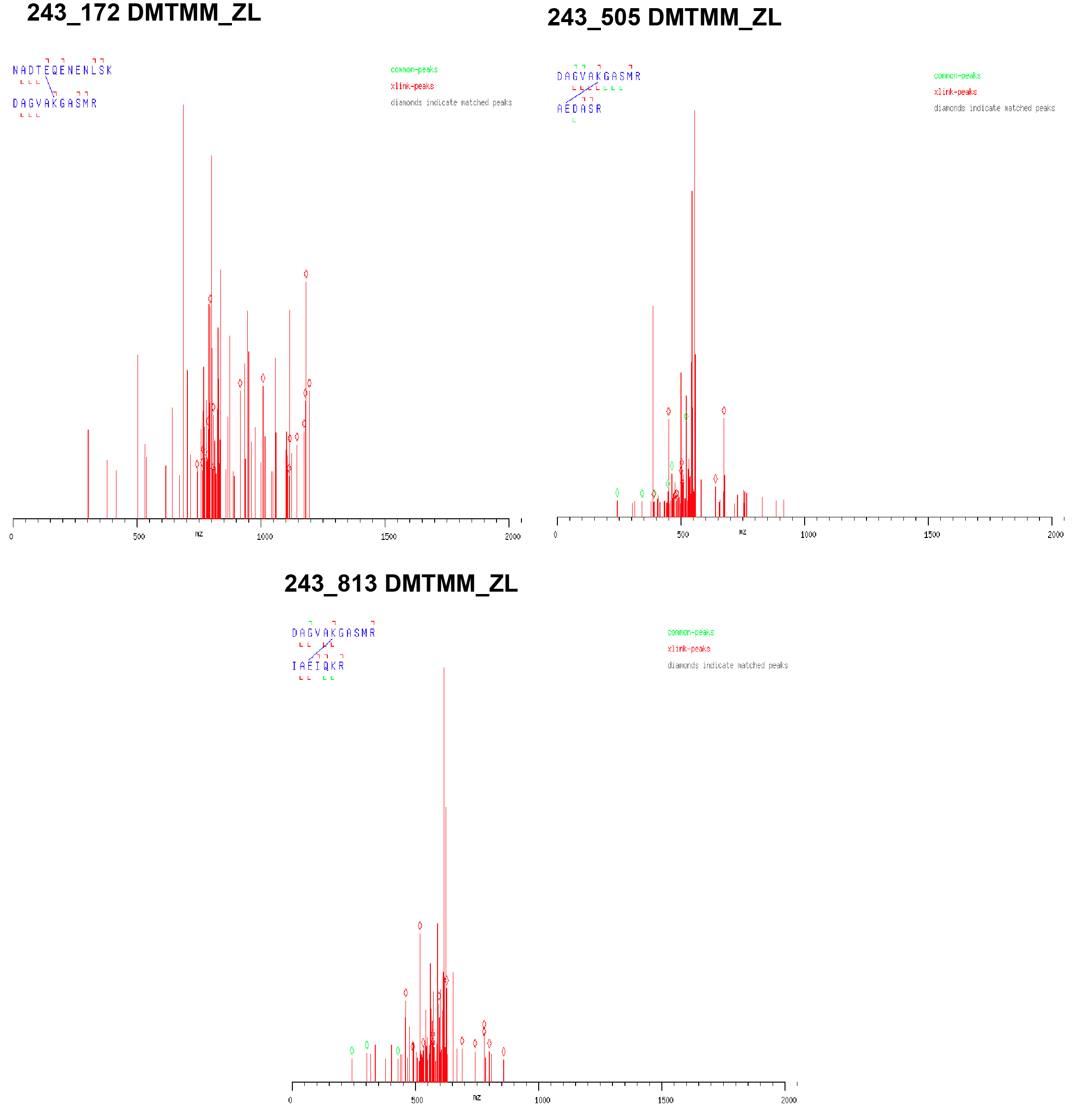


**Supplementary Figure 13. Xlink-peak MS2 spectra for crosslinks between 243-172, 243-505 and 243-813 (DMTMM_ZL) derived from PCSK9:Hsp104 complex.**


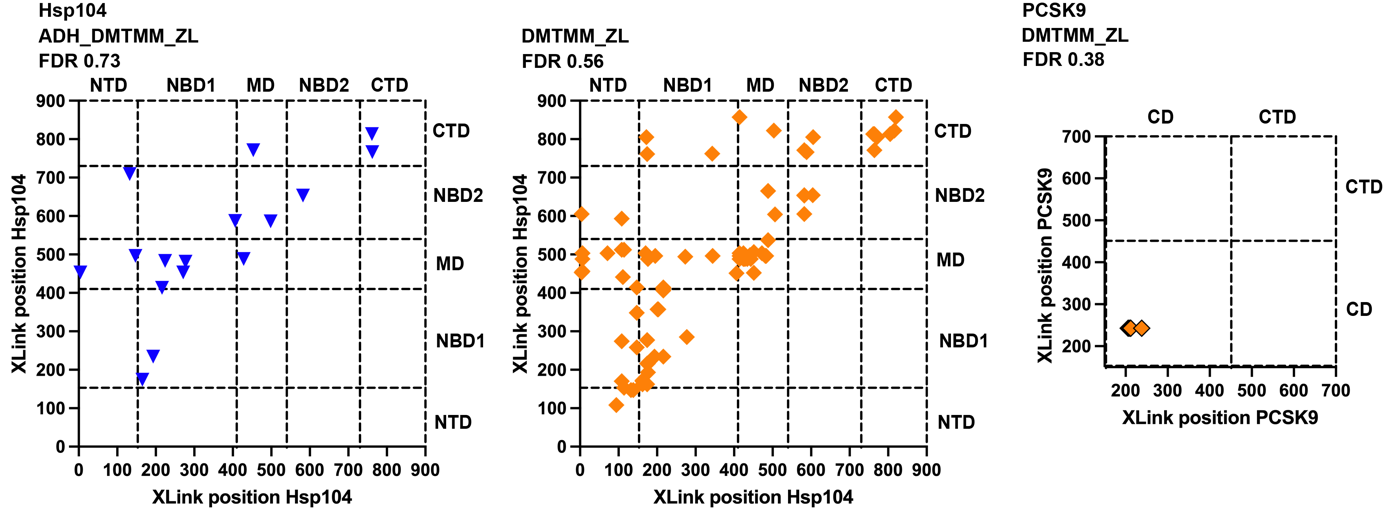


**Supplementary Figure 14. Hsp104fl and PCSK9 regions that form crosslinks.**

Crosslinked pairs plotted to show connectivity of intra contacts within and across domains from the Hsp104fl and PCSK9 XL-MS datasets. The largest number of crosslinks was formed within NBD1 and MD domains of Hsp104fl and CD region of PCSK9. Additionally, for DMTMM_ZL PCSK9 crosslinking data, it was revealed that residue 243 forms 4 crosslinks with the following residues: 206, 210, 212 and 238. For ADH_DMTMM_ZL PCSK9 no TP crosslinks were detected. Cross-links are coloured by chemistry and are shown in orange, and blue for DMTMM_ZL and ADH_DMTMM_ZL respectively. Domains are separated by dashed lines.
